# SCF^Fbxo22^-KDM4A targets methylated p53 for degradation and regulates senescence

**DOI:** 10.1038/ncomms10574

**Published:** 2016-02-12

**Authors:** Yoshikazu Johmura, Jia Sun, Kyoko Kitagawa, Keiko Nakanishi, Toshiya Kuno, Aya Naiki-Ito, Yumi Sawada, Tomomi Miyamoto, Atsushi Okabe, Hiroyuki Aburatani, ShengFan Li, Ichiro Miyoshi, Satoru Takahashi, Masatoshi Kitagawa, Makoto Nakanishi

**Affiliations:** 1 Department of Cell Biology, Graduate School of Medical Sciences, Nagoya City University, 1 Kawasumi, Mizuho-cho, Mizuho-ku, 467-8601 Nagoya, Japan; 2 Department of Molecular Biology, Hamamatsu University School of Medicine, Higashi-ku, 431-3192 Hamamatsu, Japan; 3 Department of Perinatology, Aichi Human Service Center, Institute for Developmental Research, 713-8 Kamiya-cho, Kasugai, Aichi 489-0392, Japan; 4 Department of Experimental Pathology and Tumor Biology, Graduate School of Medical Sciences, Nagoya City University, 1 Kawasumi, Mizuho-cho, Mizuho-ku, 467-8601 Nagoya, Japan; 5 Department of Comparative and Experimental Medicine and Center for Animal Sciences, Graduate School of Medical Sciences, Nagoya City University, 1 Kawasumi, Mizuho-cho, Mizuho-ku, 467-8601 Nagoya, Japan; 6 Genome Science Division, Research Center for Advanced Science and Technology, The University of Tokyo, Meguro-ku, 153-8904 Tokyo, Japan; 7 Zhongshan Hospital of Dalian University, 6 Jiefang St, Zhongshan District, 116001 Dalian, China

## Abstract

Recent evidence has revealed that senescence induction requires fine-tuned activation of p53, however, mechanisms underlying the regulation of p53 activity during senescence have not as yet been clearly established. We demonstrate here that SCF^Fbxo22^-KDM4A is a senescence-associated E3 ligase targeting methylated p53 for degradation. We find that Fbxo22 is highly expressed in senescent cells in a p53-dependent manner, and that SCF^Fbxo22^ ubiquitylated p53 and formed a complex with a lysine demethylase, KDM4A. Ectopic expression of a catalytic mutant of KDM4A stabilizes p53 and enhances p53 interaction with PHF20 in the presence of Fbxo22. SCF^Fbxo22^-KDM4A is required for the induction of p16 and senescence-associated secretory phenotypes during the late phase of senescence. *Fbxo22*^*−/−*^ mice are almost half the size of *Fbxo22*^*+/−*^ mice owing to the accumulation of p53. These results indicate that SCF^Fbxo22^-KDM4A is an E3 ubiquitin ligase that targets methylated p53 and regulates key senescent processes.

## Abstract (web summary)

Cellular senescence—the permanent cessation of cell proliferation—is a process that can be deregulated in cancer and other aging-related diseases. Here the authors demonstrate that the SCF^Fbxo22^-KDM4A complex plays an essential role during senescence as an E3 ligase that targets methylated p53 for degradation.

## Graphical abstract

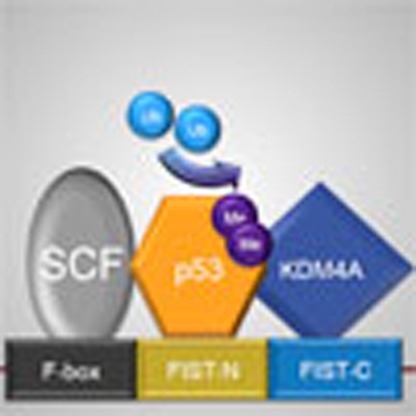

An important hallmark of senescence is the inability to proliferate in response to physiological mitotic stimuli^1^. The limited lifespan of human cells is governed by telomere length^2^,^3^ as well as various genotoxic stressors, all of which ultimately activate DNA-damage responses^4^. We and others have recently uncovered a molecular mechanism involved in permanent cell cycle arrest during the senescence process in which p53 activation at G2 has a necessary and sufficient role by inducing a mitosis skip^5^,^6^. Another hallmark of senescence is the appearance of senescence-associated secretory phenotypes (SASP), such as robust secretion of numerous growth factors, cytokines, proteases and other proteins, which can cause deleterious effects on the tissue microenvironment^7^. On the other hand, SASP also has positive effects on the repair of damaged tissue, at least at a young age^8^. Induction of these two hallmarks of senescence is often coordinated, but their respective mechanisms do not always overlap. Most notably, p38MAPK is critically required for SASP through activating NF-κB independent of canonical DDR, but p53 restrains p38MAPK leading to the suppression of SASP in senescent cells^9^. There appear to be missing links that could more fully explain the antagonistic effects of p53 on the induction of these two representative hallmarks of senescence.

The key to the regulation of p53 activity is control of the stability of its protein, which is mainly orchestrated through a network of ubiquitylation reactions^10^,^11^, although other mechanisms such as regulation of its localization are also involved^12^,^13^. While numerous E3 ubiquitin ligases for p53 have been reported^14^, data are less clear regarding the *in vivo* relevance of these E3 ligases in p53 regulation except for murine double minute 2 (Mdm2; refs 15, 16). Mdm2 is itself a transcriptional target of p53, and acts to create a negative feedback loop^17^. Importantly, in mice with a disrupted p53-Mdm2 feedback loop, the degradation profile of p53 upon DNA damage appeared to be normal^18^, suggesting the role of Mdm2 as the sole E3 ubiquitin ligase for stress-induced p53 into question.

Several lines of evidence have clearly indicated that post-transcriptional modification of p53 also has a critical role in the regulation of its activity^11^,^19^. For example, DNA-damage-induced phosphorylation of p53 at Ser15 stabilizes and activates p53, suppressing Mdm2-mediated p53 ubiquitylation^20^. Acetylation or methylation of lysine residues located at the C-terminal domain (CTD) of p53 is also reported to regulate p53 activity^21^,^22^. Although acetylation at the CTD is indispensable for p53 activation, methylation appears to vary in the degree to which it is required according to both the location and extent of the methylation state^23^. More importantly, the effect of the interplay between acetylation and methylation at the CTD of p53 is largely unknown.

Fbxo22 is not yet a well-characterized F-box protein. It was first identified as a p53-targeting gene^24^, then was later reported to form a complex with KDM4 whose degradation regulates histone H3 methylation at lysines 9 and 36 (ref. 25). Here, we identify the SCF^Fbxo22^-KDM4A complex as an E3 ubiquitin ligase for methylated p53 and show that upon senescence-inducing stimulation, SCF^Fbxo22^-KDM4A is required for induction of p16 and SASP in senescent cells.

## Results

### Fbxo22 is highly expressed in senescent cells

**Figure 1:**
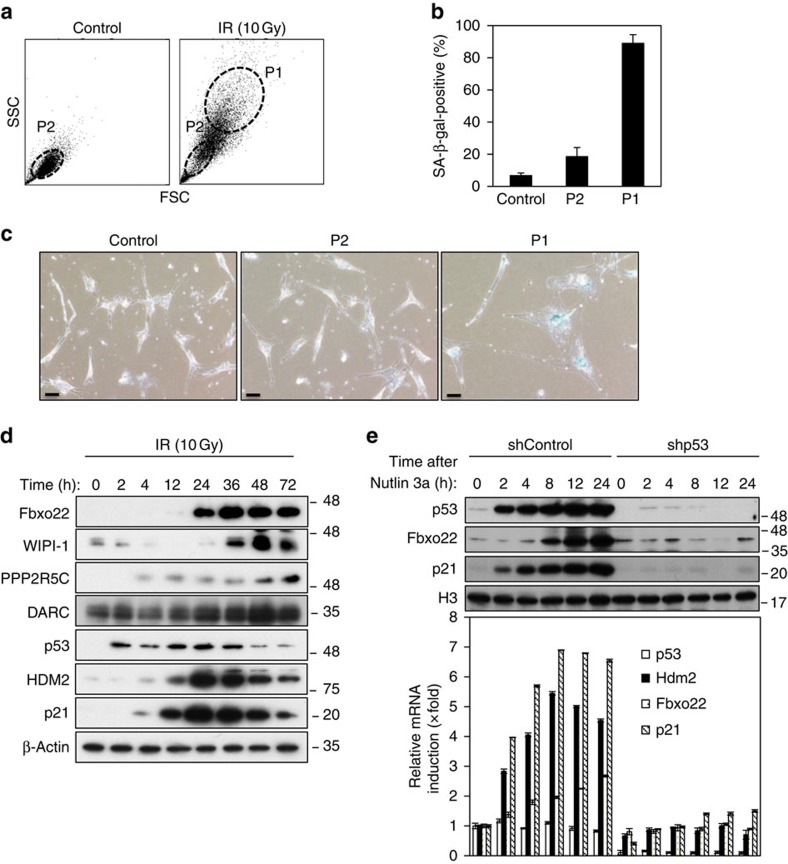
Identification of Fbxo22 as a protein predominantly expressed in larger sized senescent cells and regulation of its expression by p53. (**a**) HCA2 cells at 72 h after IR treatment (10 Gy) were sorted according to size and structure (FSC and SSC) and fractionated as P1 and P2 by FACScan. In the absence of IR (Control), the vast majority of cells were fractionated into the P2 fraction. (**b**) HCA2 cells at 72 h after IR irradiation (10 Gy) were sorted by FSC and SSC into P1 and P2 fractions as shown in Fig. 1a. The sorted cells, as well as HCA2 cells without IR irradiation as a control, were cultured for an additional 3 days, and then subjected to an SA-β-gal assay. (**c**) Representative images of cells in **b** are shown. (**d**) Lysates from HCA2 cells collected at the indicated times after IR (10 Gy) treatment were subjected to immunoblotting using the indicated antibodies. (**e**) RPE cells expressing Dox-inducible control shRNA (shControl) or p53 shRNA (shp53) were treated with doxycycline (1 μg ml^−1^). Cell lysates (upper panels) or total RNAs (lower panels) of cells collected at the indicated times after treatment with Nutlin 3a (5 μg ml^−1^) were subjected to immunoblotting using the indicated antibodies or qPCR analysis, respectively. Data are presented as means ±s.d. of at least three independent experiments.

We have recently uncovered the molecular basis of senescence induction, which results at least in part from generation of tetraploid G1 cells by mitosis skipping^5^. In order to determine the factor(s) that regulate senescent processes, we first tried to identify the genes that are predominantly expressed in larger sized senescent cells with tetraploid DNA (Fig. 1a and Supplementary Fig. 1a,b). The P1 fraction predominantly exhibited typical senescent phenotypes (SA-β-gal-positive and flattened morphology), whereas the P2 fraction did not (Fig. 1b,c). Global expression analysis using sorted larger sized cells treated with IR (10 Gy) revealed that 33 genes were expressed at levels fourfold greater than in normal-sized cells (Supplementary Fig. 1c). IR treatment of normal human fibroblast HCA2 cells revealed that Fbxo22 as well as WIPI-1, PPP2R5C, and DARC were markedly induced at relatively later time points when compared with Hdm2 (a human Mdm2 homologue) and p21 (Fig. 1d). Thus, the results suggested that our screening preferentially isolated more slowly induced genes after senescence-inducing stimulation. Induction of Fbxo22 in response to IR was dependent on the presence of p53 since p53 depletion in RPE cells almost completely abolished its induction at both RNA and protein levels (Fig. 1e). Again, induction of Fbxo22 appeared markedly slower when compared with Hdm2 and p21. Thus, these results indicated that Fbxo22 is a transcriptional target of p53 as reported previously^24^. Consistent with this, Fbxo22 possesses two p53 binding consensuses on its promoter region (Supplementary Fig. 2, left panels). ChIP-seq analysis of the region around the Fbxo22 gene confirmed recruitment of p53 to the transcription start site of this gene in the presence of 5FU in HCT116 *p53*^*+/+*^ cells, but not in the isogenic *p53*^*−/−*^ cells (Supplementary Fig. 2, right panels). This recruitment of p53 was associated with an increase in the H3K27Ac level and the extension of phospho-RNA polymerase II (P-RNAPII).

### Fbxo22 regulates the abundance of p53 and cell proliferation

**Figure 2:**
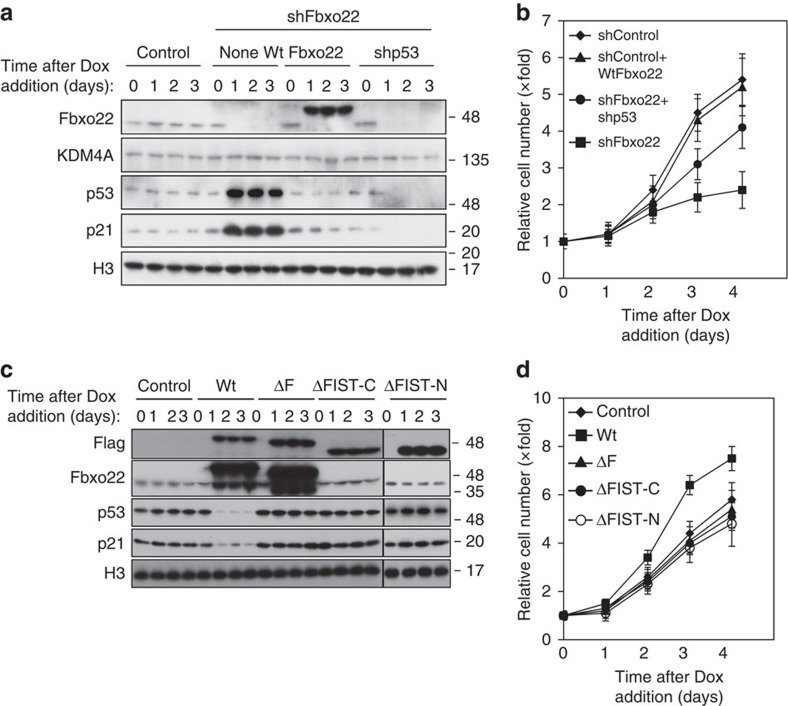
Fbxo22 controls cell proliferation through regulation of the p53 level. (**a**) RPE cells expressing the Dox-inducible shControl or shFbxo22 with or without either Dox-inducible Fbxo22 or shp53 were treated with doxycycline (1 μg ml^−1^). Lysates of cells collected at the indicated times were subjected to immunoblotting using the indicated antibodies. (**b**) The relative numbers of cells indicated as in **a** were determined at the indicated times. (**c**) RPE cells expressing Dox-inducible wild-type Fbxo22 (Wt), its mutants lacking the F-box domain (ΔF), FIST-C (ΔFIST-C) or FIST-N (ΔFIST-N), as well as control RPE cells (Control), were treated with doxycycline (1 μg ml^−1^). Lysates of cells collected at the indicated times were subjected to immunoblotting using the indicated antibodies. Because of the antibody epitope, Fbxo22 antibodies failed to recognize ΔFIST-C and ΔFIST-N proteins. (**d**) The relative numbers of cells as in **c** were determined at the indicated times. Data are presented as means ±s.d. of at least three independent experiments.

When RPE cells were depleted of Fbxo22 using doxycycline-inducible Fbxo22 shRNA, the levels of p53 and p21 were significantly increased (Fig. 2a). These inductions were effectively suppressed by re-introduction of wild-type Fbxo22, eliminating the possibility that the influence of Fbxo22 depletion resulted from its off-target effect. Although KDM4A was reported to be targeted for proteosomal degradation by Fbxo22 (ref. 25), the level of KDM4A was not affected in RPE cells by Fbxo22 depletion independent of the p53 status (Fig. 2a). Fbxo22 depletion resulted in a severe defect in cell proliferation, which was effectively recovered by re-introduction of wild-type Fbxo22 or simultaneous depletion of p53 (Fig. 2b). The impaired cell proliferation appeared to be owing to accumulation of cells at G1 phase (Supplementary Fig. 3a, four left panels). In contrast, overexpression of Fbxo22 resulted in the loss of p53 and p21 (Fig. 2c). These effects were not observed when its F-box (ΔF), FIST-C (ΔFIST-C) or FIST-N (ΔFIST-N) domain-deleted mutants were expressed. Hence, overexpression of wild-type Fbxo22, but not ΔF, ΔFIST-C or ΔFIST-N mutants, enhanced cell proliferation (Fig. 2d) owing to the reduction in the population of G1 cells (Supplementary Fig. 3a, four right panels). Although treatment with Nutlin 3a markedly induced p53, p21 and Hdm2 in RPE cells, ectopic Fbxo22 strongly suppressed their respective inductions even under inhibition of Hdm2 or Hdm2 depletion (Supplementary Fig. 3b,c), indicating that the suppression of p53 by Fbxo22 is independent of Mdm2. Immunohistochemical analyses revealed that this protein predominantly localized in the nucleus (Supplementary Fig. 3d).

### SCF^Fbxo22^ ubiquitylates p53

**Figure 3:**
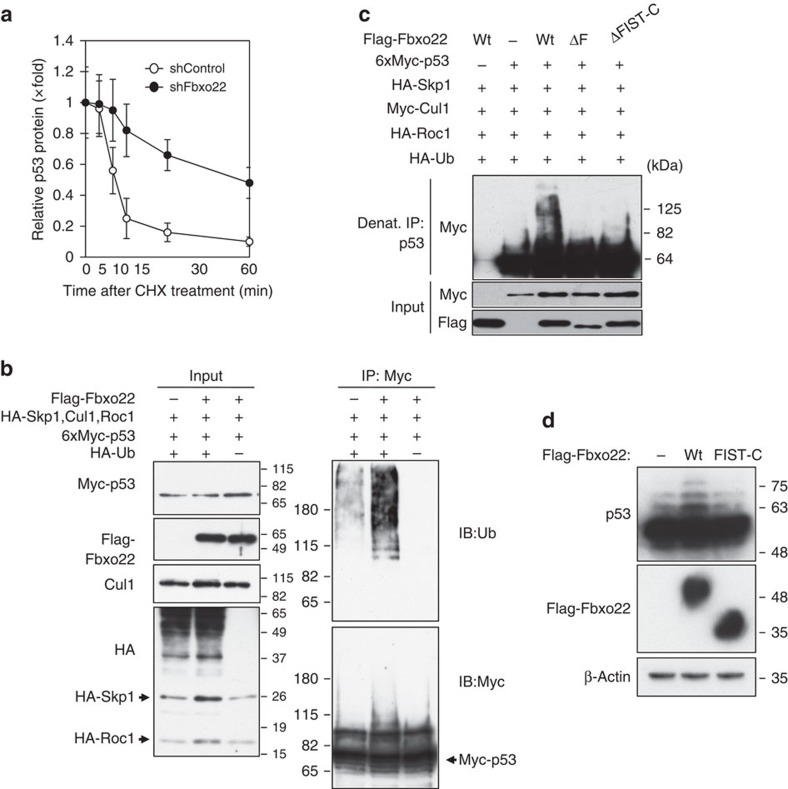
SCF^Fbxo22^ ubiquitylates p53. (**a**) RPE cells expressing the Dox-inducible shControl or shFbxo22 in the presence of doxycycline (1 μg ml^−1^) for 48 h were treated with 50 μg ml^−1^ cycloheximide (CHX). Lysates of cells collected at the indicated times were subjected to immunoblotting using the indicated antibodies and the relative p53 intensities were determined using Image J. Data are presented as means±s.d. of at least three independent experiments. (**b**) U2OS cells were transfected with the indicated expression plasmids including SCF components, and were then incubated with MG132 (20 μg ml^−1^) for 5 h. Total cell lysates were subjected to immunoprecipitation with the indicated antibodies under native conditions. The resultant precipitates and lysates (Input) were subjected to immunoblotting using the indicated HRP-conjugated antibodies. (**c**) To prevent the detection of ubiquitylation of both E3 ligase itself and p53-associated proteins, the cell lysates were immunoprecipitated with anti-p53 antibody under denaturing conditions. The resultant immunoprecipitates and lysates (Input) were subjected to immunoblotting using the indicated HRP-conjugated antibodies. (**d**) RPE cells expressing the Dox-inducible full-length Flag-Fbxo22 (Wt) or its mutant lacking the FIST-C domain (FIST-C), as well as control RPE cells were incubated in the presence of doxycycline (1 μg ml^−1^) for 48 h and then treated with MG132 (10 μg ml^−1^) for 6 h. Cell lysates were subjected to immunoblotting using the indicated antibodies.

Cycloheximide treatment revealed that the half-life of p53 appeared to be around 10 min in control RPE cells, whereas it was around 60 min in Fbxo22-depleted RPE cells (Fig. 3a). Thus, Fbxo22 likely regulates p53 stability under unperturbed conditions. Indeed, treatment with the proteasome inhibitor, MG132, almost completely abolished Fbxo22-dependent reduction of p53 protein (Supplementary Fig. 4a). Using non-denatured extracts, a greatly enhanced ubiquitylation signal was detected on p53 in the presence of Fbxo22 (Fig. 3b). Similarly, under denatured IP conditions, a strong ubiquitylation signal was detected in the presence of wild-type Fbxo22, but not its mutants lacking the F-box or FIST-C domain (Fig. 3c and Supplementary Fig. 4b). Consistent with this, ectopic expression of wild-type Fbxo22, but not its mutant lacking FIST-C, facilitated the ubiquitylation of p53 in RPE cells (Fig. 3d). In order to investigate whether Fbxo22 could mediate K48-linked polyubiquitylation (a major form of degradation-associated ubiquitylation^26^), we transfected HEK293 cells with plasmids expressing a mutant ubiquitin in which lysine 48 was replaced by arginine (K48R) or in which all lysines were replaced except for lysine 48 in a ubiquitin molecule (K48) as well as plasmids expressing 6xMyc-p53 and Flag-Fbxo22. Cells were then treated with MG132 and the ubiquitylation of p53 was analysed. The Fbxo22-mediated ubiquitylation signal was readily detected in cells expressing K48 ubiquitin but not in those expressing K48R (Supplementary Fig. 4c). Thus, our results suggest that SCF^Fbxo22^ mainly promotes K48-linked ubiquitylation of p53. Taken together, these results indicate that SCF^Fbxo22^ acts as an E3 ubiquitin ligase in targeting p53 for degradation.

### Fbxo22 binds to the unacetylated CTD of p53

**Figure 4:**
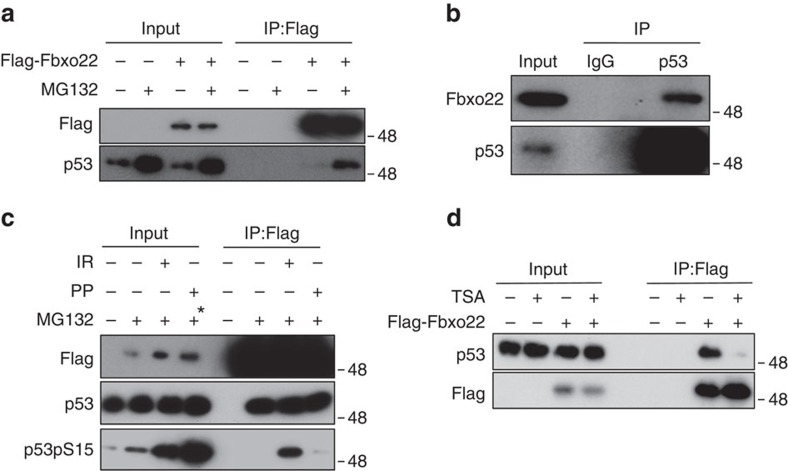
p53 interacts with Fbxo22 in a phosphorylation-independent manner. (**a**) RPE cells expressing Dox-inducible Flag-Fbxo22 were incubated for 48 h in the presence or absence of doxycycline (1 μg ml^−1^) and were then treated with MG132 (10 μg ml^−1^) for 2 h. The cell lysates were immunoprecipitated using anti-Flag M2 affinity gel. The resultant immunoprecipitates and lysates (Input) were subjected to immunoblotting using anti-Flag or anti-p53 antibodies. (**b**) RPE cells were treated with MG132 (10 μg ml^−1^) for 2 h and the lysates were subjected to immunoprecipitation using the anti-p53 antibody or a control IgG. The resultant immunoprecipitates and lysates (Input) were subjected to immunoblotting using the indicated antibodies. (**c**) Phosphorylation of p53 did not affect its interaction with Fbxo22. RPE cells expressing Dox-inducible Flag-Fbxo22 were incubated for 48 h in the presence or absence of doxycycline (1 μg ml^−1^) and were then treated with or without MG132 (10 μg ml^−1^) for 2 h and/or with IR (6 Gy). The lysates were immunoprecipitated using anti-Flag M2 affinity gel. After immunoprecipitation, some samples were treated with protein phosphatase (PP) for 1 h. The resultant immunoprecipitates and lysates (Input) were subjected to immunoblotting using the indicated antibodies. The asterisk indicates an input sample before PP treatment. (**d**) Interaction between Fbxo22 and p53 was suppressed by treatment with TSA. RPE cells expressing Dox-inducible Flag-Fbxo22 were incubated for 48 h in the presence or absence of doxycycline (1 μg ml^−1^) and were then treated with or without TSA (1 mM) for 12 h and/or MG132 (10 μg ml^−1^) for 2 h. The cell lysates were immunoprecipitated using anti-Flag M2 affinity gel. The resultant immunoprecipitates as well as lysates (Input) were subjected to immunoblotting using the indicated antibodies.

**Figure 5:**
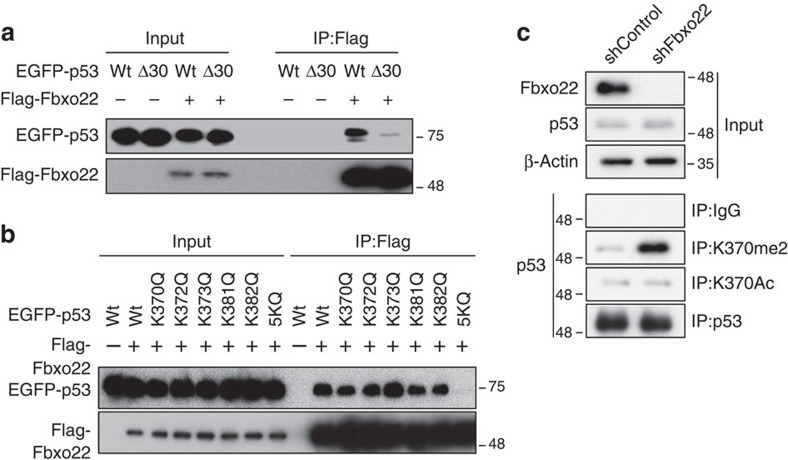
Acetylation of lysines at the CTD of p53 suppresses the interaction with Fbxo22. (**a**) *p53*^*−/−*^ HCT116 cells expressing Dox-inducible Flag-Fbxo22 together with wild-type EGFP-p53 (Wt) or a mutant p53 lacking the C-terminal 30 amino acids (Δ30) were incubated for 48 h in the presence or absence of doxycycline (1 μg ml^−1^) and were then treated with MG132 (10 μg ml^−1^) for 2 h. The lysates were immunoprecipitated using anti-Flag M2 affinity gel. The resultant immunoprecipitates and lysates (Input) were subjected immunoblotting using the indicated antibodies. (**b**) *p53*^*−/−*^ HCT116 cells expressing Dox-inducible Flag-Fbxo22 together with the wild-type or the indicated mutants of EGFP-p53 were incubated for 48 h in the presence or absence of doxycycline (1 μg ml^−1^) and were then treated with MG132 (10 μg ml^−1^) for 2 h. The lysates were immunoprecipitated using anti-Flag M2 affinity gel. The resultant immunoprecipitates and lysates (Input) were subjected to immunoblotting using the indicated antibodies. (**c**) RPE cells expressing the Dox-inducible shControl or shFbxo22 were incubated for 48 h in the presence of doxycycline (1 μg ml^−1^) and were then treated with MG132 (10 μg ml^−1^) for 2 h. The lysates were immunoprecipitated using a control IgG, methyl (K370me2)- or acetyl (K370Ac)-specific anti-p53, or anti-p53 antibodies. The resultant immunoprecipitates and lysates (Input) were subjected to immunoblotting using the indicated antibodies.

Ectopically expressed Flag-Fbxo22 in RPE cells formed a stable complex with p53 in the presence of MG132, whereas this complex was not detected in the absence of MG132 (Fig. 4a). Endogenous Fbxo22 also formed a complex with p53 in RPE cells when treated with MG132 (Fig. 4b). Although the F-box protein often interacts with its substrates through a phosphorylation-dependent degron^27^ and multiple sites of p53 are phosphorylated in response to DNA damage^20^, Fbxo22 equally bound to p53 treated with or without IR and phosphatase (Fig. 4c). Interestingly, complex formation between Fbxo22 and p53 was significantly suppressed when RPE cells were treated with an HDAC inhibitor, trichostatin A, suggesting that acetylation of p53 inhibited their interaction (Fig. 4d). Since most acetylation sites of p53 are located at the CTD, we speculated whether Fbxo22 could bind to the CTD of p53. As expected, Fbxo22 effectively bound to wild-type p53, but not to its CTD-deleted mutant (Δ30; Fig. 5a). In order to further confirm that acetylation of p53 CTD prevents complex formation between Fbxo22 and p53, we generated mutants harbouring an acetyl-mimic substitution (K to Q) at each or all lysines on the CTD and tested their abilities to bind to Fbxo22. Although the K to Q substitution at each lysine residue did not affect p53 binding to Fbxo22, the substitution at all lysines (5KQ) almost completely abolished their interactions (Fig. 5b). Interestingly, the K to A substitution, but not the K to Q substitution, at all five lysines as well as at two lysine sites at which p53 was methylated did not affect their interactions, suggesting that substitution at these lysines did not result in marked conformational changes in the p53 CTD domain. These results also suggest that Fbxo22 likely bound to the methylated p53 but failed to bind to the acetylated p53 (Supplementary Fig. 5). We then examined the effect of methylation at these lysines on Fbxo22-dependent degradation of p53. Intriguingly, methylation of p53 at K370 was greatly increased in Fbxo22-depleted RPE cells in the presence of MG132, whereas acetylation at this site was not (Fig. 5c). Taken together, the results strongly suggest that SCF^Fbxo22^ preferentially targeted methylated p53, but not acetylated p53, in ubiquitin-dependent degradation.

### SCF^Fbxo22^ forms a ternary complex with p53 and KDM4A

**Figure 6:**
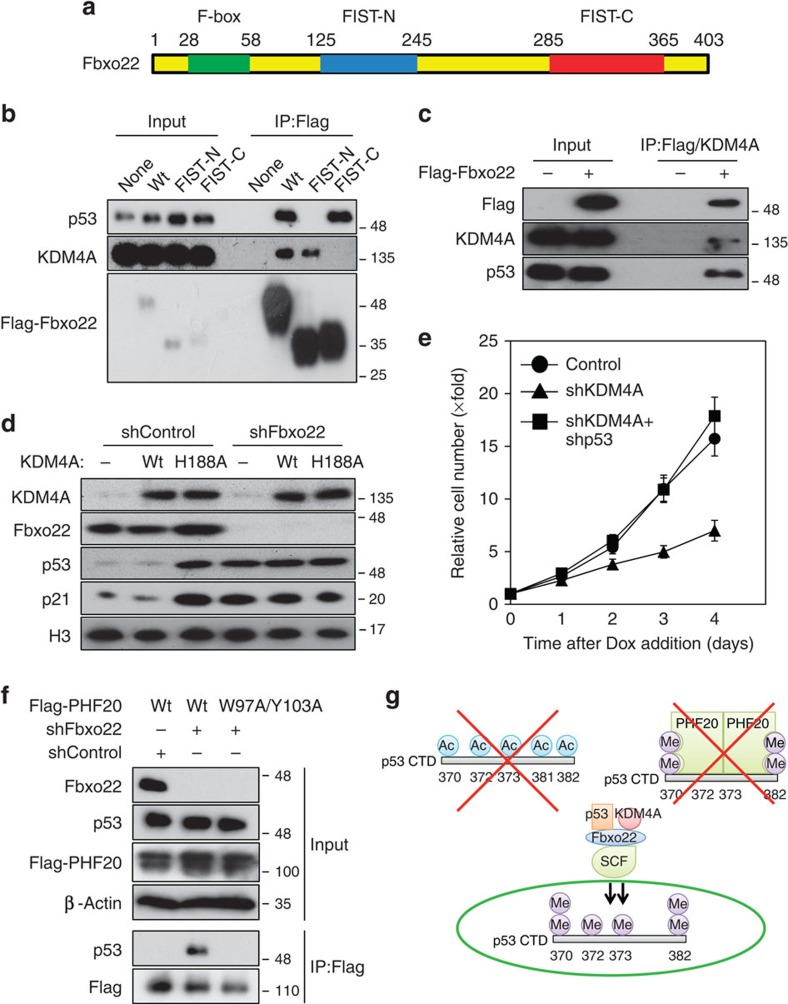
SCF^Fbxo22^ forms a ternary complex with p53 and KDM4A that targets methylated p53 for degradation. (**a**) Schematic representation of the domain structure of Fbxo22. (**b**) RPE cells expressing Dox-inducible wild-type Flag-Fbxo22 (Wt), and its mutants lacking FIST-N (ΔFIST-N) or FIST-C (ΔFIST-C) were incubated for 48 h in the presence or absence of doxycycline (1 μg ml^−1^) and were then treated with MG132 (10 μg ml^−1^) for 2 h. The lysates were immunoprecipitated using anti-Flag M2 affinity gel. The resultant immunoprecipitates and lysates (Input) were subjected to immunoblotting using the indicated antibodies. (**c**) RPE cells expressing Dox-inducible Flag-Fbxo22 were incubated for 48 h in the presence or absence of doxycycline (1 μg ml^−1^) and were then treated with MG132 (10 μg ml^−1^) for 2 h. The lysates were subjected to sequential immunoprecipitation using anti-Flag M2 affinity gel and anti-KDM4A antibodies. The resultant immnoprecipitates and lysates were subjected to immunoblotting using the indicated antibodies. (**d**) RPE cells expressing the Dox-inducible shControl or shFbxo22 together with wild-type KDM4A (Wt) or its catalytically inactive mutant (H188A) were incubated for 48 h in the presence of doxycycline (1 μg ml^−1^) and were then treated with MG132 (10 μg ml^−1^) for 2 h. The lysates were subjected to immunoblotting using the indicated antibodies. (**e**) RPE cells expressing the Dox-inducible shControl, shKDM4A or shKDM4A+shp53 were treated with doxycycline (1 μg ml^−1^) and their relative numbers were determined at the indicated times. Data are presented as means±s.d. of at least three independent experiments. (**f**) RPE cells expressing the Dox-inducible shControl or shFbxo22, together with wild-type Flag-PHF20 (Wt) or its WD domain mutant (W97A/Y103A) were incubated for 48 h in the presence of doxycycline (1 μg ml^−1^) and were then treated with MG132 (10 μg ml^−1^) for 2 h. The cell lysates were immunoprecipitated using anti-Flag M2 affinity gel. The resultant immunoprecipitates and lysates (Input) were subjected to immunoblotting using the indicated antibodies. (**g**) Schematic representation of CTD modifications that protect p53 from SCF^Fbxo22^-targeted degradation.

Fbxo22 contains F-box, FIST-N and FIST-C domains (Fig. 6a). p53 bound to the wild type and to a mutant lacking FIST-C, but not to a mutant lacking FIST-N (Fig. 6b), indicating that FIST-N of Fbxo22 is a p53-binding site for this protein. Given that previous mass spectrometry revealed that Fbox22 formed a stable complex with KDM4A^25^ and that SCF^Fbxo22^ preferentially targeted methylated p53, we speculated that SCF^Fbxo22^ might form a ternary complex with p53 and KDM4A. Wild-type Fbxo22 and its mutant lacking FIST-N bound to KDM4A, whereas the mutant lacking FIST-C failed to do so (Fig. 6b), indicating that the FIST-C domain of Fbxo22 is a KDM4A-binding site. Fbxo22 interacted with the SCF complex through its F-box domain, showing that the N-terminal portion of Fbxo22 (1–100) was sufficient for the interaction with Skp1 (Supplementary Fig. 6a). In order to confirm that Fbxo22, p53 and KDM4A form a ternary complex, we performed sequential immunoprecipitation using an anti-Flag M2 affinity gel, followed by elution using Flag peptide and then carried out a second immunoprecipitation using anti-KDM4A antibodies. p53 was readily detectable in Flag/KDM4A immunoprecipitates (Fig. 6c). Importantly, the interaction between p53 and KDM4A required Fbxo22 (Supplementary Fig. 6b), indicating that Fbxo22 functions as a scaffold for p53 and KDM4A.

We then asked whether KDM4A demethylase activity is required for SCF^Fbxo22^-dependent p53 destabilization. Ectopic expression of wild-type KDM4A did not cause p53 to accumulate in RPE cells, whereas that of a mutant KDM4A lacking demethylase activity (H188A) markedly increased the p53 level (Fig. 6d). This increase was not affected by Fbxo22 depletion, although its depletion *per se* increased the amount of p53 to a level similar to that in cells expressing the mutant KDM4A. In addition, expression of the mutant KDM4A (H188A), but not the wild type, resulted in a specific increase in methylated, but not acetylated, p53 in the presence of MG132, suggesting that KDM4A likely demethylates p53 (Supplementary Fig. 6c). Taken together, SCF^Fbxo22^-mediated p53 degradation requires the demethylase activity of KDM4A. Consistent with this, depletion of KDM4A in RPE cells resulted in a severe defect in cell proliferation (Fig. 6e), owing to the increase in the expression levels of p53 and p21, because co-depletion of p53 effectively reversed impaired cell proliferation in KDM4A-depleted cells (Supplementary Fig. 6d).

Recently, PHF20 was reported to bind to doubly dimethylated p53, stabilizing and activating p53 (ref. 28). When Fbxo22 was depleted from RPE cells, the interaction between ectopically expressed Flag-PHF20 and p53 was greatly enhanced (Fig. 6f). This interaction was completely abolished when two amino acids within the Tudor domain of PHF20 were replaced with alanine. Furthermore, when the demethylase-deficient mutant of KDM4A was expressed, the interaction between PHF20 and p53 was greatly enhanced (Supplementary Fig. 7a). The enhanced interaction was not further affected by Fbxo22 depletion (Supplementary Fig. 7b), indicating that KDM4A functions together with Fbxo22. Depletion of PHF20 suppressed induction of p53 and p21 on IR treatment (Supplementary Fig. 7c). Taken together, these results strongly suggest that SCF^Fbxo22^-KDM4A targets methylated p53 for ubiquitin-dependent protein degradation. Acetylation of p53 and PHF20 interaction with dimethylated p53 compete with this ubiquitylation (Fig. 6g).

### Fbxo22 is required for induction of p16 and SASP

**Figure 7:**
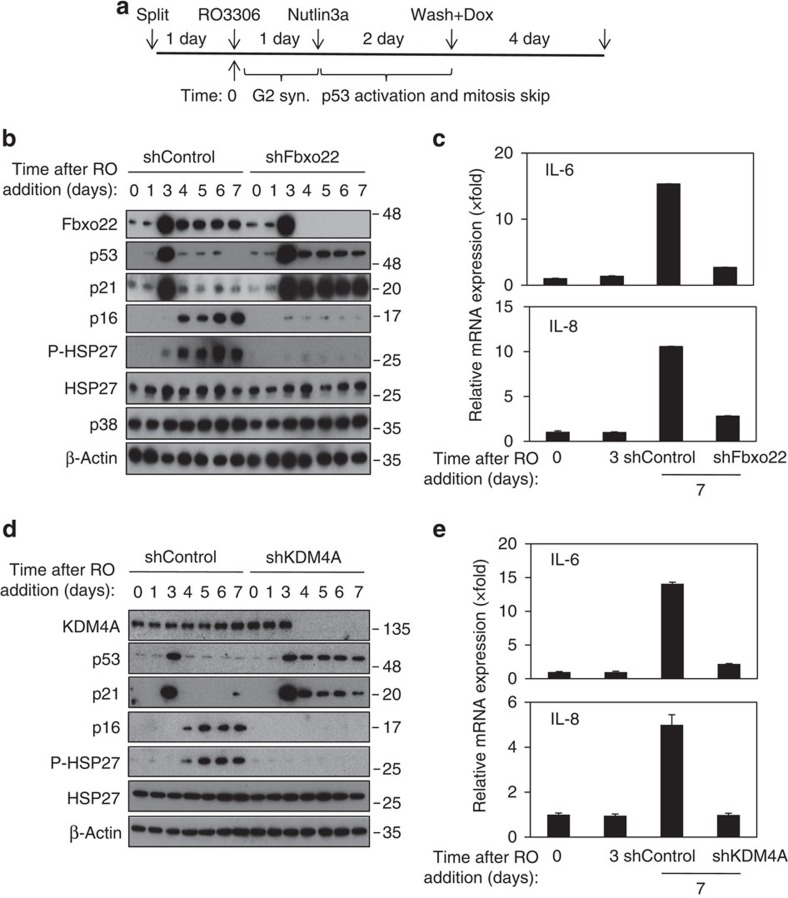
SCF^Fbxo22^-KDM4A-dependent degradation of p53 is essential for induction of p16 and SASP in senescent cells. (**a**) Experimental outline of Fbxo22 or KDM4A depletion in senescent cells. (**b**) HCA2 cells expressing the Dox-inducible shControl or shFbxo22 treated as in **a** were subjected to immunoblotting using the indicated antibodies. (**c**) Total RNAs from HCA2 cells as in **b** were subjected to 7 days of qPCR analysis using the indicated primers. (**d**) HCA2 cells expressing the Dox-inducible shControl or shKDM4A treated as in **a** were subjected to immunoblotting using the indicated antibodies. (**e**) Total RNAs from HCA2 cells as in **d** were subjected to 7 days of qPCR analysis using the indicated primers. Data are presented as means±s.d. of at least three independent experiments.

We then determined the physiological relevance of the negative feedback between p53 and SCF^Fbxo22^-KDM4A in senescence. For this purpose, we induced senescence by specific activation of p53 at G2 phase because this protocol does not cause DNA damage and activate DNA-damage responses, which allowed us to analyse the senescence process more specifically. When Fbxo22 was depleted according to the experimental outline (Fig. 7a), p53 and p21 were readily detectable after removal of Nutlin 3a, whereas they were hardly detectable in control cells (Fig. 7b). Treatment with Nutlin 3a at G2 phase according to this experimental outline effectively induced senescence phenotypes such as an increased 4 N population, an increased SA-β-gal-positive cells, an increased ROS level and cessation of cell proliferation without inducing apoptosis and DNA damage (Supplementary Fig. 8a–f). Induction of p16 and activation of p38MAPK, as evaluated by HSP27 phosphorylation, were greatly suppressed in Fbxo22-depleted Nutlin 3a-induced senescent cells although Fbxo22 depletion did not affect most of the senescent phenotypes such as an increased 4 N population, an increased in SA-β-gal-positive cells, a rise in the ROS level, and cessation of cell proliferation induced by this experimental outline (Supplementary Fig. 9a–d). Accordingly, induction of IL-6 and IL-8 was strongly compromised in Fbxo22-depleted cells, but not in control cells (Fig. 7c). A requirement for Fbxo22 in the induction of p16 and SASP was also observed in replicative senescent cells (Supplementary Fig. 10a–c) as well as in oncogene-induced senescent cells (Supplementary Fig. 11a–c). In addition, similar results were also observed in KDM4A-depleted Nutlin 3a-induced senescent cells (Fig. 7d,e). Again, KDM4A depletion did not affect most of the senescent phenotypes (Supplementary Fig. 12a–d). The suppression of p16 and SASP induction in senescent cells by continuous activation of p53 was confirmed by continuous treatment with Nutlin 3a (Supplementary Fig. 13a,b) or Hdm2 depletion (Supplementary Fig. 14a–c). Once again, continuous activation of p53 did not affect most of the senescent phenotypes, including cessation of cell proliferation, an increased ROS level, and an increased in SA-β-gal-positive cells, whereas it strongly suppressed NF-κB activity which is reported to be essential for SASP^9^,^29^ (Supplementary Fig. 8a–g). As expected, expression of wild-type, but not W97A/Y103A mutant, PHF20 in Nutlin 3a-induced senescent cells compromised p53 reduction and suppressed p16 and SASP induction, although their expression did not affect most of the senescent phenotypes (Supplementary Fig. 15a–e). Thus, a negative feedback loop between p53 and Fbxo22 plays an important role in induction of p16 and SASP in senescent cells. The SCF^Fbxo22^-KDM4A complex appears to be a central player in the induction of senescence phenotypes.

### Fbxo22 regulates the abundance of p53 *in vivo*

**Figure 8:**
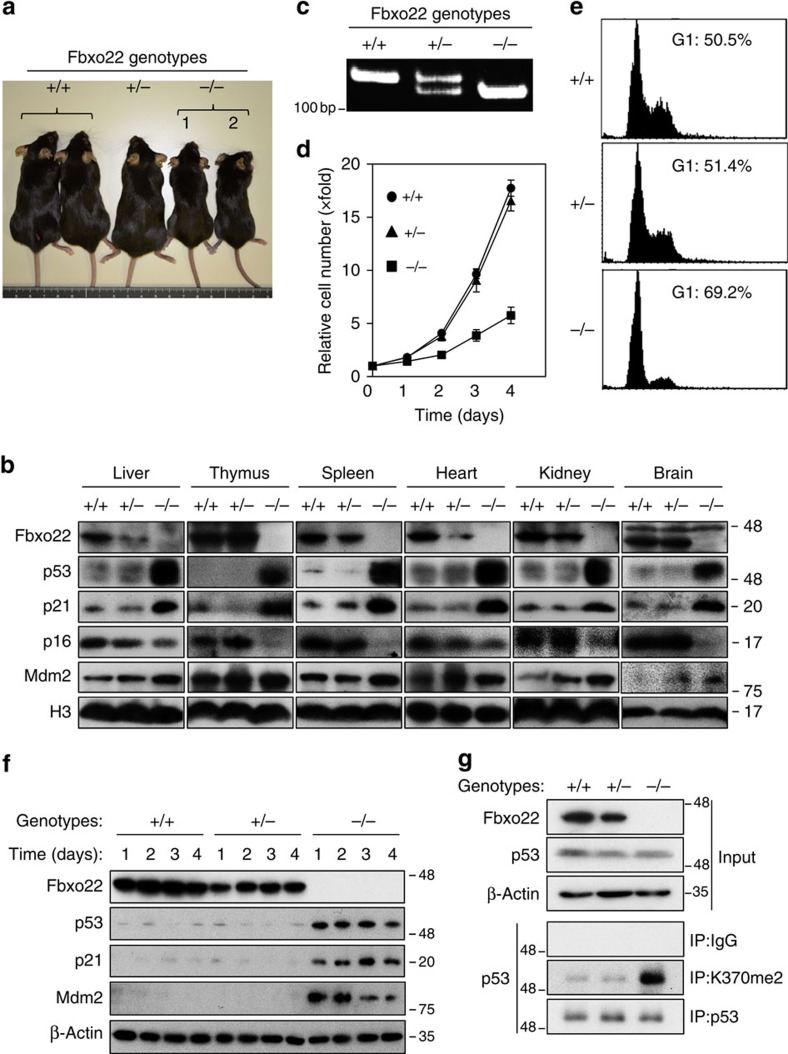
CRISPR/CAS9-mediated disruption of mouse Fbxo22. (**a**) Representative *Fbxo22* wild-type (+/+), heterozygous (+/−) or nullizygous (−/−) mice at 24 weeks of age. (**b**) Lysates of tissues from the spleen, thymus, kidney, liver, lung and brain of 24-week-old *Fbxo22* wild-type (+/+), heterozygous (+/−) or nullizygous (−/−) mice were analysed by immunoblotting using the indicated antibodies. (**c**) Genotype analysis of *Fbxo22* wild-type (+/+), heterozygous (+/−) and nullizygous (−/−) mice by PCR. (**d**) Primary MEFs from *Fbxo22*^*−/−*^ mice showed severe growth retardation. Relative numbers of primary MEFs from *Fbxo22*^*+/+*^, *Fbxo22*^*+/−*^ and *Fbxo22*^*−/−*^ mice were determined at the indicated times. Data are presented as means±s.d. of at least three independent experiments. (**e**) Cell cycle distribution of primary MEFs as in **d** was determined at 4 days by FACScan. (**f**) Primary MEFs from *Fbxo22*^*−/−*^ mice showed marked accumulations of p53, p21 and Mdm2. Primary MEFs from mice with the indicated genotypes were collected at the indicated times and the lysates were subjected to immunoblotting as shown. (**g**) Primary MEFs with the indicated genotypes were treated with MG132 (10 μg ml^−1^) for 2 h. The lysates were immunoprecipitated using a control IgG, methyl (K370me2)-specific anti-p53 or anti-p53 antibodies. The resultant immunoprecipitates and lysates (Input) were subjected to immunoblotting using the indicated antibodies.

In order to clarify the physiological function of Fbxo22 in p53 regulation, we generated Fbxo22 mutant mice using the CRISPR-Cas9 system^30^. Pronuclear injection of circular plasmids expressing Cas9 and single-guide RNA that targets exon 1 of the Fbxo22 gene resulted in generation of 3 nullizygous (*Fbxo22*^*−/−*^) and one heterozygous mouse (*Fbxo22*^*+/−*^), all of which possessed distinct deletions or mutations in exon 1 of the Fbxo22 gene (Supplementary Fig. 16a). Although one *Fbxo22*^*+/−*^ mouse appeared normal, two *Fbxo22*^*−/−*^ mice were viable but were of smaller size with almost half the body weight at 6 months of age as compared with *Fbxo22*^*+/−*^ or *Fbxo22*^*+/+*^ mice (Fig. 8a). Relative organ weights of these *Fbxo22*^*−/−*^ mice were comparable with those of *Fbxo22*^*+/−*^ or *Fbxo22*^*+/+*^ mice (Supplementary Fig. 16b). The other *Fbxo22*^*−/−*^ mouse died within 2 days of birth. In order to eliminate the possibility that the phenotypes observed in *Fbxo22*^*−/−*^ mice were owing to off-target effects *in vivo*, we amplified DNA fragments by PCR from all 9 potential off-target loci (Supplementary Fig. 17) predicted by a previously determined recognition rule^30^, and found no mutations, deletions or insertions. Taken together with the fact that all mutant mice possessed distinct mutations or deletions, phenotypes in *Fbxo22*^*−/−*^ mice were unlikely to have been because of the off-target effects. We intercrossed *Fbxo22*^*+/−*^ mice and found that the genotype distribution of the offspring was consistent with the Mendelian-based ratio of 1:2:1 although some portion of the *Fbxo22*^*−/−*^ mice died within a couple of days, indicating that, unlike *Mdm2*, *Fbxo22* is dispensable for early embryonic development.

p53 and p21 as well as Mdm2 markedly accumulated in all tissues tested in *Fbxo22*^*−/−*^ mice (Fig. 8b). Interestingly, the level of p16 in these mice was greatly decreased in all tissues tested, indicating that the role of SCF^Fbxo22^-KDM4A in the induction of p16 was also conserved *in vivo*. Histo-pathological analyses of tissues from *Fbxo22*^*−/−*^ mice revealed that their anatomical structures appeared normal (data not shown). Mouse embryonic fibroblast (MEFs) from *Fbxo22*^*−/−*^ mice showed a severe growth defect when compared with those from *Fbxo22*^*+/+*^ or *Fbxo22*^*+/−*^ mice (Fig. 8c,d), owing to the increase in the G1 population (Fig. 8e). As in mice tissues, p53, p21 and Mdm2 markedly accumulated in MEFs from *Fbxo22*^*−/−*^, but not *Fbxo22*^*+/+*^ and *Fbxo22*^*+/−*^ mice (Fig. 8f). In addition, methylated p53 was significantly increased in MEFs from Fbxo22^−/−^ mice, but not Fbxo22^+/+^ or Fbxo22^+/−^ mice in the presence of MG132 (Fig. 8g). Ectopically expressed wild-type PHF20, but not its W97A/Y103A mutant, formed a complex with p53 in cells from *Fbxo22*^*−/−*^ mice, but not *Fbxo22*^*+/+*^ and *Fbxo22*^*+/−*^ mice (Supplementary Fig. 18), reflecting the increase in the amount of methylated p53 in cells from *Fbxo22*^*−/−*^ mice. These results clearly show that the phenotypes observed in *Fbxo22*^*−/−*^ mice were consistent with those in their MEFs.

## Discussion

The functioning of p53 in senescence processes is biphasic; it is essential in the early phase of senescence in mitotic skipping by its phase-specific activation at G2 phase, whereas it must be removed at the late phase for the induction of p16 and SASP to occur. This suggests that the pathways regulating the level and timing of p53 activation are critical for overall senescence regulation. In this study, we showed that the SCF^Fbxo22^-KDM4A complex is an indispensable element through its function as a novel E3 ubiquitin ligase complex targeting methylated p53 for degradation at the late senescent stage.

Over a dozen E3 ubiquitin ligases and modulators have been reported to regulate p53 stability under certain circumstances. However, there is overwhelming molecular and genetic evidence supporting a major role for Mdm2 in targeting p53 for degradation under both stress and non-stress conditions^10^,^11^,^14^ but the *in vivo* relevance of other E3 ligases in p53 regulation is far less clear. In this respect, we successfully identified the SCF^Fbxo22^-KDM4A complex as a *bona fide* E3 ligase for p53. The strongest evidence for this is that target disruption of *Fbxo22* in mice and their MEFs resulted in a marked increase in amounts of p53, p21 and Mdm2, but a decrease in p16 (Fig. 8a,b), which resulted in the small body size of these mice and the severe growth defects affecting their MEFs (Fig. 8c–f). Similar phenotypes were observed in *Skp2*^*−/−*^ mice and MEFs in which another Cdk inhibitor, p27, had accumulated^31^. Similar to Mdm2, the *Fbxo22* gene is a direct transcriptional target of p53, suggesting a plausible mechanism by which p53 activity could be reset to basal levels after shutdown of p53-inducible stimuli, although its p53-dependent induction was relatively slower than that of Mdm2 and p21 (Fig. 1d,e and Supplementary Fig. 2). Taken together, the normal p53 degradation profile of mice with a disrupted p53-mdm2 negative feedback loop may be explained in terms of SCF^Fbxo22^-p53 circuitry.

SCF^Fbxo22^ bound to the CTD of p53 (Fig. 5a), and unlike Mdm2, this binding was not affected by p53 phosphorylation induced by DNA damage (Fig. 4c). In contrast, SCF^Fbxo22^ binding to p53 was affected by acetylation and/or methylation of the lysine cluster at K370–K386 on p53 CTD^19^. Given that acetylated p53 was not a degradation target of SCF^Fbxo22^-KDM4A (Fig. 5b), this could effectively explain why acetylation on the p53 CTD by CBP/p300 greatly increased p53 stabilization^32^. Although this increased stability might also be reflected by competitive modifications between CBP/p300-mediated acetylation and Mdm2-mediated ubiquitylation at the CTD^33^, this scenario is unlikely because methylation at K372 or K370 by KMT5 or KMT3C did not increase p53 stabilization^34^. In this respect, depletion of Fbxo22 in cells resulted in a specific increase in the abundance of methylated p53, but not acetylated p53 (Fig. 5c) and overexpression of a catalytic mutant, KDM4A, resulted in p53 stabilization (Fig. 6d). Thus, the SCF^Fbxo22^-KDM4A complex likely targets methylated p53 for ubiquitin-dependent degradation, consistent with previous reports showing that most p53 methylation acted as a mark of repression of p53-dependent transcriptional activity^19^. In addition, it should be noted that KDM4A was reported to suppress senescence by negatively regulating the p53 pathway via transcriptional suppression of CHD5 (ref. 35). Our results are also consistent with a recent report that PHF20 leads to p53 stabilization and activation^28^ because PHF20 binding to methylated p53 protected it from SCF^Fbxo22^-KDM4A-mediated degradation (Fig. 6f). Taken together, the interplay between acetylation and methylation at the p53 CTD directly regulate p53 stability by controlling the access of Fbxo22 to the p53 CTD (Fig. 6g).

Although p53 is essential for triggering senescence induction, subsequent induction of p16 appears critical for maintaining irreversible growth arrest^5^,^36^. p53 was reported to inhibit p38MAPK, leading to the suppression of SASP phenotypes^9^. Thus, p53 must be downregulated at the late stage of senescence, demonstrating the biphasic functioning of p53. Fine-tuning of this biphasic function is likely achieved by temporal orchestration of p53 ubiquitylation by Mdm2 and SCF^Fbxo22^-KDM4A which regulate early and late events, respectively. Most notably, SCF^Fbxo22^-KDM4A is essential for SASP induction and could therefore be a promising candidate for the development of drugs targeting cancer as well as geriatric diseases, since cytokines and growth factors secreted from senescent cells have recently been implicated in chronic inflammation and various age-related changes, including carcinogenesis *in vivo*^7^,^37^,^38^.

## Methods

### Cell culture

Early passage HCA2 (ref. 39), HeLa (ATCC), U2OS (ATCC), MCF7 (RIKEN Cell Bank) cells, MEFs and HEK-293T (RIKEN Cell Bank) cells were cultured in DMEM (Invitrogen) supplemented with 10% fetal bovine serum (FBS). RPE-1 (ATCC) were cultured in DMEM/F-12 (Invitrogen) supplemented with non-heat-inactivated 10% FBS. HCT116 cells were grown in McCoy's 5A (Gibco) supplemented with FBS. Cells were synchronized at G2 phase by treatment with 9 μM of RO3306 (Roche) for 24 h. Nutlin 3a (Sigma-Aldrich), cycloheximide (Sigma-Aldrich), trichostatin A or MG132 (Sigma-Aldrich) was used at a concentration of 5 μM, 50 μg ml^−1^, 1 mM or 10 μg ml^−1^, respectively, for the specified intervals.

### Plasmid construction

To generate lentivirus-based shRNA constructs, a 19–21 bp shRNA-coding fragment with a 5′-ACGTGTGCTGTCCGT-3′ loop was introduced into pENTR4-H1 digested with *Age*I/*Eco*RI. To insert the H1tetOx1-shRNA into lentivirus vector, we mixed the resulting pENTR4-H1-shRNA vector and CS-RfA-ETBsd vector with Gateway LR clonase (Invitrogen). All the target sequences for lentivirus-based sh-RNAs are summarized in Supplementary Table 1. To construct Tet-on-inducible lentivirus constructs, the PCR-generated *Bam*HI/*Not*I fragments containing cDNA for human/mouse Fbxo22 wild type or the respective mutants, human KDM4A wild type or H188A and human p53 wild type or the respective mutants, were inserted into a pENTR-1A vector (Invitrogen) containing the Flag epitope or EGFP digested with *BamH*I/*Not*I. The resultant plasmid was mixed with CS-IV-TRE-RfA-UbC-Puro or CS-IV-TRE-RfA-UbC-Hygro vector, and reacted with Gateway LR clonase to generate the lentivirus plasmid. Plasmids expressing *hCas9* and sgRNA were prepared by ligating oligos for the targeting site (mouse Fbxo22 exon1-163-185) into the BbsI site of pX330. The pcDNA3-HA-KDM4A, pcDNA-3-FLAG-PHF20 and pX330 plasmids were kindly provided by Dr Kristian Helin, Dr Mark Bedford and Feng Zhang, respectively.

### FACS analysis and FACS sorting

For FACS sorting, HCA2 cells treated with IR (10 Gy) were sorted according to size and structure (FSC and SSC) using a BD FACSAria-2 cell sorter (BD Biosciences). For cell cycle profiles, cells were collected and fixed with 70% ethanol. Cells were then washed once with phosphate-buffered saline (PBS), treated with RNase and stained with propidium iodide. Flow cytometry was performed using a FACS CANTO2 flow cytometer (BD Biosciences).

### Microarray analysis

Total RNA was prepared using ISOGEN II (Nippongene). RNA hybridization, washing and analysis were performed using SurePrint G3 Human GE 8 × 60 k version 2.0 (Agilent Technologies) and GeneSpring GX version 12.6.0 (Agilent Technologies).

### Virus generation and infection

Lentiviruses expressing the respective shRNAs or genes were generated by co-transfection of 293T cells with pCMV-VSV-G-RSV-RevB, pCAG-HIVgp and the respective CS-RfA-ETBsd, CS-RfA-ETHygro, CS-RfA-ETPuro, CS-IV-TRE-RfA-UbC-Puro or CS-IV-TRE-RfA-UbC-Hygro using the calcium phosphate co-precipitation method. Cells infected with the indicated viruses were treated with 10 μg ml^−1^ of blasticidin (Invitrogen), and/or 2 μg ml^−1^ of puromycin (Sigma-Aldrich) for 2–3 days. Doxycycline (Sigma-Aldrich) was added to the medium at a concentration of 1 μg ml^−1^ for inducible expression of the respective shRNAs or genes.

### Immunoprecipitation and immunoblotting analyses

Cells were lysed in TBSN buffer (20 mM Tris-Cl (pH 8.0), 150 mM NaCl, 0.5% NP-40, 5 mM EGTA, 1.5 mM EDTA, 0.5 mM Na_3_VO_4_ and 20 mM *p*-nitrophenylphosphate). The resulting lysates were clarified by centrifugation at 15,000*g* for 20 min at 4 °C before immunoprecipitation with the specified antibody. For whole lysates, cells or tissues were directly lysed with Laemmli-buffer (2% SDS, 10% glycerol, 5% 2-mercaptoethanol, 0.002% bromophenol blue and 62.5 mM Tris-HCl at pH 6.8). The whole lysates (20–50 μg) were separated by SDS-polyacrylamide gel electrophoresis (SDS-PAGE), transferred to a polyvinylidene difluoride(*Immobilon-*P; Millipore) membrane, and then subjected to immunoblotting with the indicated antibodies using the enhanced chemiluminescence-detection system. All antibodies used in this study are listed in Supplementary Table 2. Full scans of the most important blots are provided in the Supplementary Fig. 19.

### Quantitative RT-PCR

Total RNA was extracted using ISOGEN II (Wako) according to the manufacturer's instructions. For qRT-PCR analysis, cDNAs were synthesized using a SuperScript II cDNA synthesis kit (Invitrogen). Real-time PCR amplifications were performed in 96-well optical reaction plates with Power SYBR Green PCR Master Mix (Applied Biosystems). The relative expression values of each gene were determined by normalization to GAPDH expression for each sample. Primer sequences: p53-Fw (5′-TCAACAAGATGTTTTGCCAACTG-3′), p53-Rv (5′-ATGTGCTGTGACTGCTTGTAGATG-3′), p21-Fw (5′-TCAGGGTCGAAAACGGCG-3′), p21-Rv (5′-AAGATCAGCCGGCGTTTGGA-3′), Fbxo22-Fw (5′-CTCACTGAAGTAGGTCTTTTAG-3′), Fbxo22-Rv (5′-CCAGCCAAGATGATATTCATATC-3′), Hdm2-Fw (5′-ACCTCACAGATTCCAGCTTCG-3′), Hdm2-Rv (5′-TTTCATAGTATAAGTGTCTTTTT-3′), GAPDH-Fw (5′-GAGTCAACGGATTTGGTC GT-3′), GAPDH-Rv (5′-TTGATTTTGGAGGGATCTCG-3′), IL-6-Fw (5′-CCAGGAGCCCAGCTATGAAC-3′), IL-6-Rv (5′-CCCAGGGAGAAGGCAACTG-3′), IL-8-Fw (5′-AAGGAAAACTGGGTGCAGAG-3′), IL-8-Rv (5′-ATTGCATCTGGCAACCCTAC-3′)

### SA-β-gal staining

SA-β-gal staining was performed as previously described^40^. Cells were washed in PBS, fixed for 5 min in 2% formaldehyde/0.2% glutaraldehyde, washed and incubated at 37 °C with fresh SA-β-gal stain solution (1 mg of X-gal per ml/40 mM citric acid/sodium phosphate, pH 6.0/5 mM potassium ferrocyanide/5 mM potassium ferricyanide/150 mM NaCl/2 mM MgCl_2_) for 24 h. After staining, cells were washed in PBS, fixed in methanol for 3 min and then washed in PBS.

### ChIP-sequence analysis of the Fbxo22 promoter

Cells were fixed in 1% formaldehyde for 10 min at room temperature, followed by 5 min quenching with 125 mM glycine. Cells were washed twice with PBS and cell pellets were flash frozen in liquid nitrogen. The frozen crosslinked pellets were stored at −80 °C. Cell pellets were resuspended in sonication buffer (10 mM Tris-HCl, pH 7.5, 150 mM NaCl, 1 mM EDTA, 1% SDS) and sonicated for 5 cycles of 1 min each on ice. Sepharose and magnetic beads were coupled to 2 μg of the indicated antibodies. Sonicated lysates were incubated overnight at 4 °C with the antibody-bound beads. Beads were washed once with IP dilution buffer (20 mM Tris-HCl, pH 7.5, 150 mM NaCl, 1 mM EDTA, 1% Triton X-100), once with wash buffer 1 (20 mM Tris-HCl, pH 7.5, 500 mM NaCl, 2 mM EDTA, 1% Triton X-100, 0.1% SDS) and once with wash buffer 2 (10 mM Tris-HCl, pH 7.5, 0.25 M LiCl, 1 mM EDTA, 0.5% Na-Deoxycholate, 0.5% NP-40). The reversal of crosslinkage (65 °C overnight in the presence of Proteinase K), was followed by RNase A treatment and DNA was purified by phenol–chloroform extraction and ethanol precipitation. ChIP-seq libraries for Illumina sequencing were prepared following the Illumina Truseq ChIP sample Prep Kit protocol. ChIP libraries were sequenced using an Illumina GAII sequencer for 36 bases in single-read mode.

### ChIP-seq data processing

High-throughput sequencing of the ChIP fragments was performed using an Illumina Genome Analyzer (Illumina) following the manufacturer's protocol. Sequences were mapped to the human reference genome (NCBI Build 36, hg18) using ELAND software (Illumina). As a result, 26,730,379, 17,020,836, 22,559,531, 8,296,440, 9,319,944, 29,174,994, 27,258,981, 15,964,489, 15,142,757, 27,522,467, 24,557,266, 9,486,765 and 11,280,300 reads were uniquely mapped for p53, K4me3 (5FU 0, 9 h), K4me1 (5FU 0, 9 h), K27ac (5FU 0, 9 h), Pol2 (5FU 0, 9 h), Pol2 (5FU 0, 9 h), *p53*^*−/−*^ K27ac (5FU 0, 9 h) and *p53*^*−/−*^ Pol2 (5FU 0, 9 h), respectively. The ChIP signal values (ChIP counts/expected) were generated as described^41^.

### Ubiquitination assay

Using previously described methods^42^, with some modifications, plasmids were transiently transfected into U2OS cells using X-tremeGENE 9. After 43 h, cells were treated with 20 μM MG132 for 5 h, and subsequently lysed in lysis buffer (50 mM Tris-HCl, pH 7.5, 300 mM NaCl and 0.5% Triton X-100) containing protease inhibitors and deubiquitinase inhibitor. Total cell lysates were immunoprecipitated with antibodies against anti-Myc followed by immunoblotting with HRP-conjugated antibodies against anti-Myc or anti-Ubiquitin. Moreover, to prevent the detection of ubiquitylation of both E3 ligase itself and p53-associated proteins, a ubiquitylation assay was also performed under denaturing conditions. According to the previous methods, cell lysates from transfected cells were lysed with lysis buffer and added to an equal volume of 2 × SDS-PAGE sample buffer and incubated at 100 °C for 8 min before being incubated with an anti-p53 antibody and protein G-Sepharose 4FF at 4 °C. Immunoprecipiated samples were analysed by immunoblotting with HRP-conjugated anti-Myc antibody to detect ubiquitylated p53.

### Generation of Fbxo22 knock-out mice

Four-week-old C57BL/6N female mice were superovulated and mated with 7-week-old C57BL/6N males, and the fertilized eggs were collected from the oviducts. The pronuclear stage eggs were injected with pX330-mFbxo22-4 plasmid at 5 ng μl^−1^. The eggs were cultivated in KSOM overnight, then transferred into the oviducts of 5-week-old pseudopregnant ICR females. Potential off-target sites were found using Optimized CRISPR design software ( http://crispr.mit.edu/). The ∼500 bp genomic fragments containing the target or off-target in the center were PCR-amplified and sequenced. All experimental procedures conformed to the Regulations for Animal Experimentation at Nagoya City University, and were reviewed by the Institutional Laboratory Animal Care and Use Committee of Nagoya City University and finally approved by the provost. Primary MEFs were derived from E14.5 embryos of 12-week-old double-heterozygote breeders.

## Additional information

**Accession codes:** Microarray expression profiling was performed by Oncomics (Nagoya, Japan), GEO accession: GSE69116.

**How to cite this article:** Johmura, Y. *et al*. SCF^Fbxo22^-KDM4A targets methylated p53 for degradation and regulates senescence. *Nat. Commun.* 7:10574 doi: 10.1038/ncomms10574 (2016).

## Supplementary Material

Supplementary Information Supplementary Figures 1-19 and Supplementary Tables 1-2
