# Supplementary material for: SCFFbxo22-KDM4A targets methylated p53 for degradation and regulates senescence
Source: Nat Commun. 2016 Feb 12;7:10574. doi: 10.1038/ncomms10574 (PMC4754341; doi:10.1038/ncomms10574)
Supplement: Supplementary Information — Supplementary Figures 1-19 and Supplementary Tables 1-2 [file ncomms10574-s1.pdf]

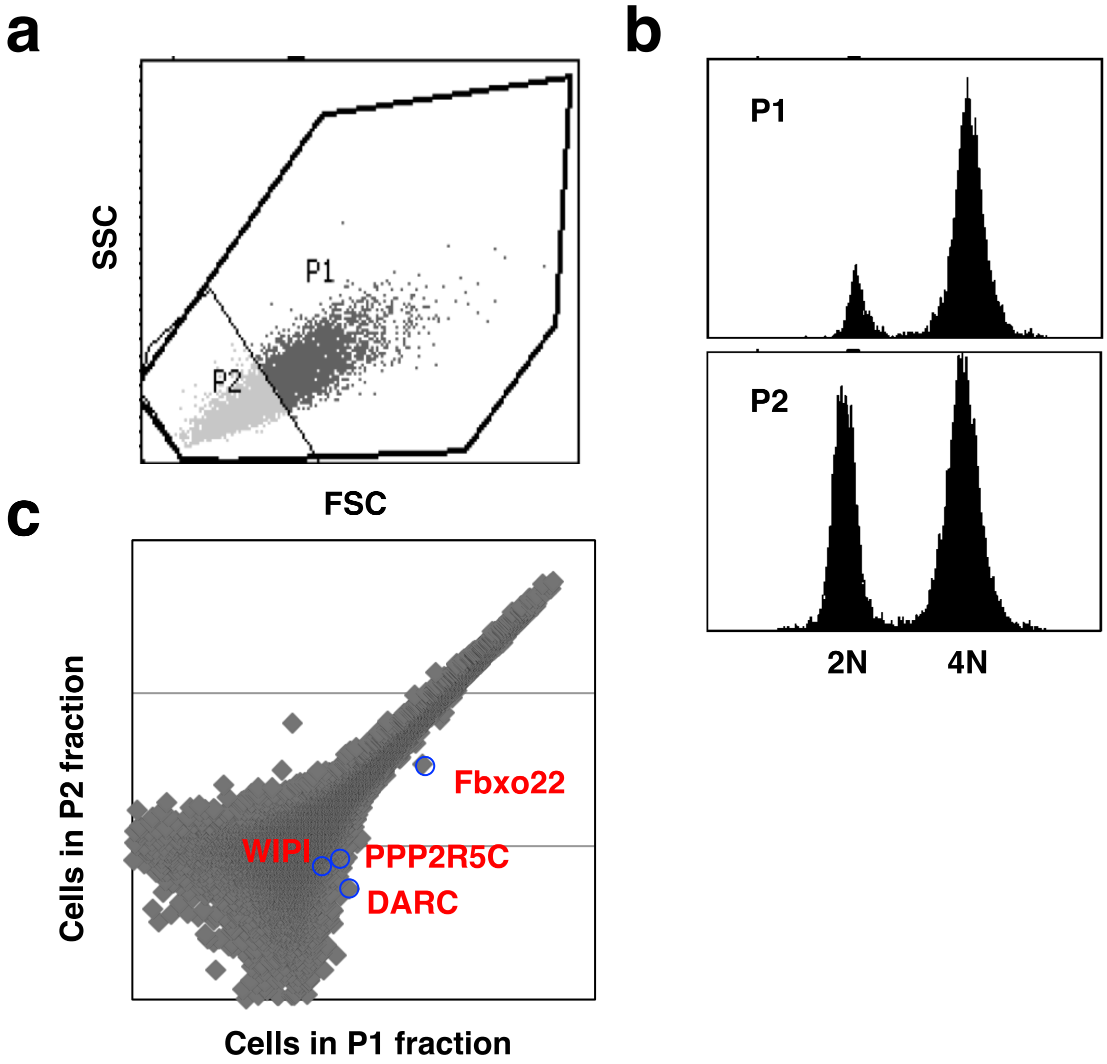


**Supplementary Figure 1 Fbxo22 predominantly expresses in larger sized tetraploid HCA2 cells after IR treatment**

**(a)** HCA2 cells at 72 hrs after IR treatment (10 Gy) were stained with Hoechst 33342 and sorted according to size and structure (FSC and SSC) into two fractions, P1 (larger sized cells) and P2 (smaller sized cells), by FACScan. **(b)** Cell cycle distributions of P1 and P2 fractions were analyzed by FACScan. **(c)** HCA2 cells at 72 hrs after IR treatment (10 Gy) were sorted by FSC and SSC as in **(a)** without staining with Hoechst 33342. Total RNAs from the P1 or P2 fraction were then subjected to microarray analysis. Scatter plots identified Fbxo22, WIPI, PPP2R5C, and DARC as genes that were significantly more abundant in the P1 fraction than in the P2 fraction. We confirmed that the four respective proteins were markedly induced after IR treatment. (see Figure 1c).

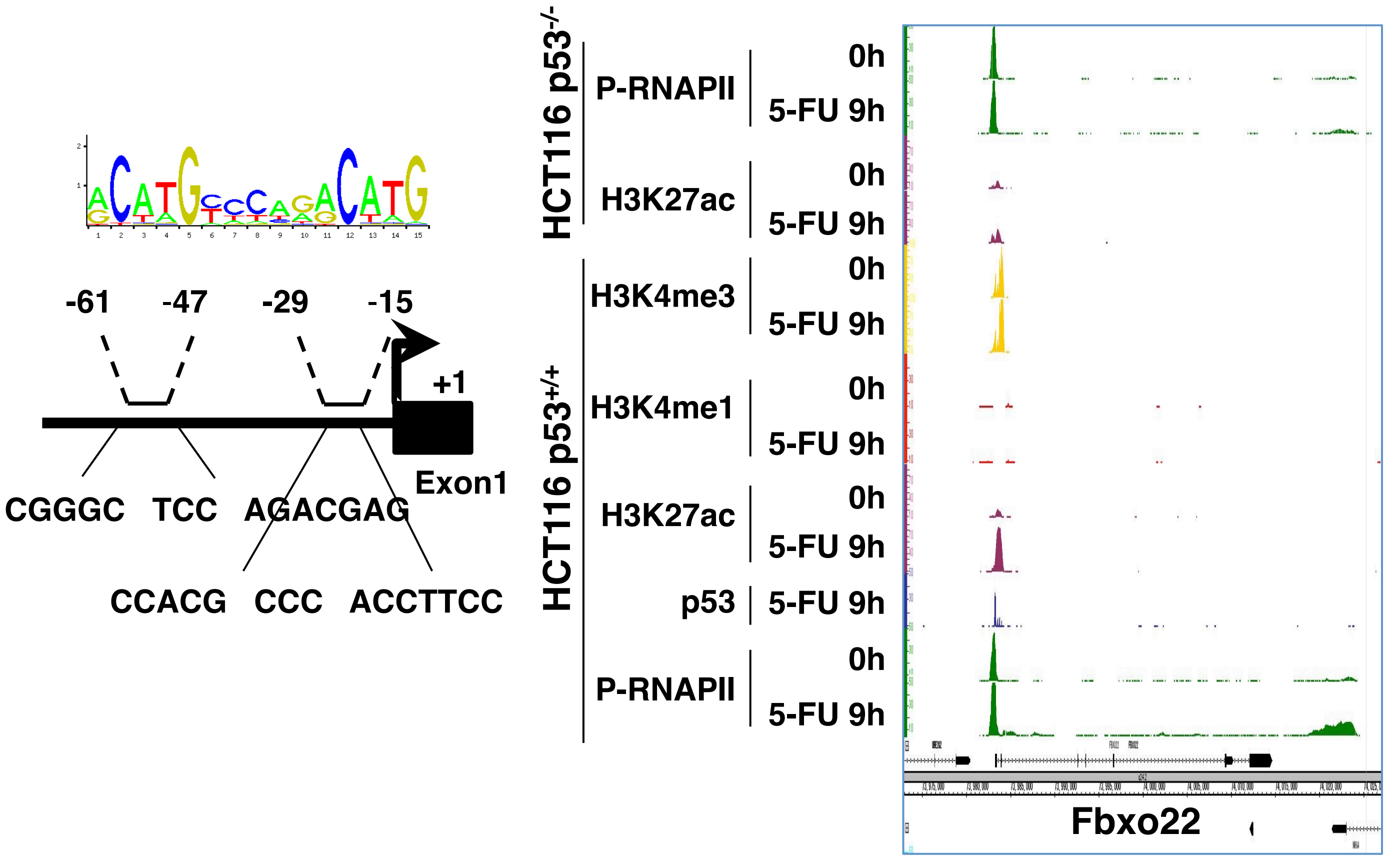


**Supplementary Figure 2 p53 regulates Fbxo22 expression**

Schematic presentation of putative p53 binding sites in the Fbxo22 promoter region identified by the JASPAR program (left panels). ChIP-seq was performed using the indicated antibodies in HCT116 $p53^{+/+}$ and HCT116 $p53^{-/-}$ cells treated with 5-FU. The genomic locus of Fbxo22 is shown together with the results obtained (right panels).

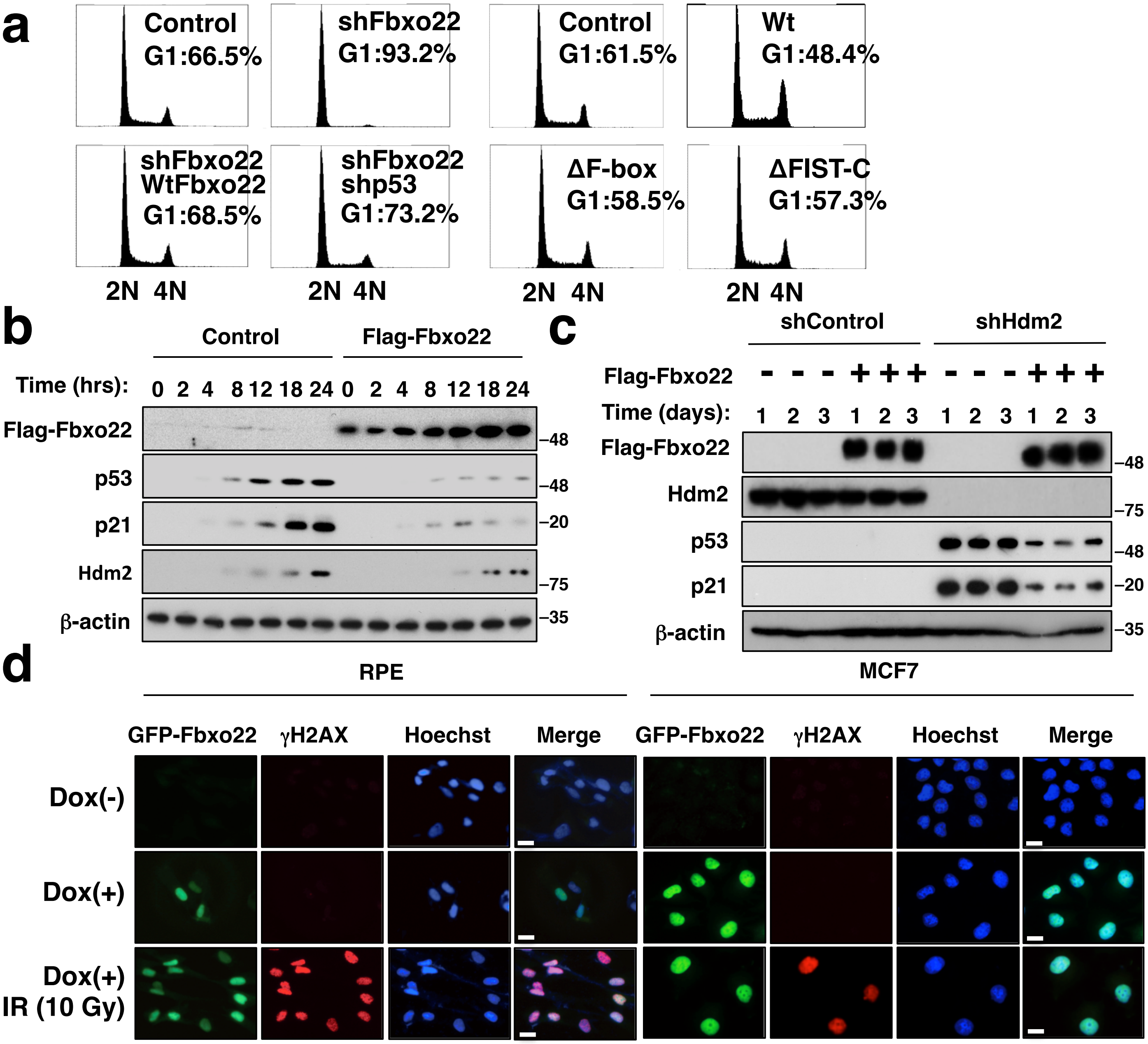


**Supplementary Figure 3 Ectopic expression of Fbxo22 results in reduced expression of p53 independent of Hdm2 and induction of G1**

**(a)** RPE cells treated as in Figure 2a (four left panels) and 2c (four right panels) at 3 days after addition of doxycycline were stained with Hoechst 33342 and analyzed for cell cycle distribution by FACScan. **(b)** RPE cells expressing Dox-inducible Flag-Fbxo22 were treated with (Flag-Fbxo22) or without (Control) doxycycline (1 μg/ml) in the presence of Nutlin 3a (5 μM). Cells were lysed at the indicated times after the addition of Nutlin 3a and the lysates were subjected to immunoblotting using the indicated antibodies. **(c)** RPE cells expressing the Dox-inducible shControl and shHdm2 together with or without Flag-Fbxo22 were treated with doxycyclin (1 μg/ml). Cells were lysed at the indicated times after the addition of doxycycline and the lysates were subjected to immunoblotting using the indicated antibodies. **(d)** RPE or MCF7 cells expressing Dox-inducible EGFP-Fbxo22 were treated with (+) or without (-) doxycycline (1 μg/ml) and/or IR (10 Gy) for 24 hrs. Cells were then immunostained using anti-EGFP or anti-γH2AX antibodies. DNA was counterstained with Hoechst 33342. Scale bars, 20μm

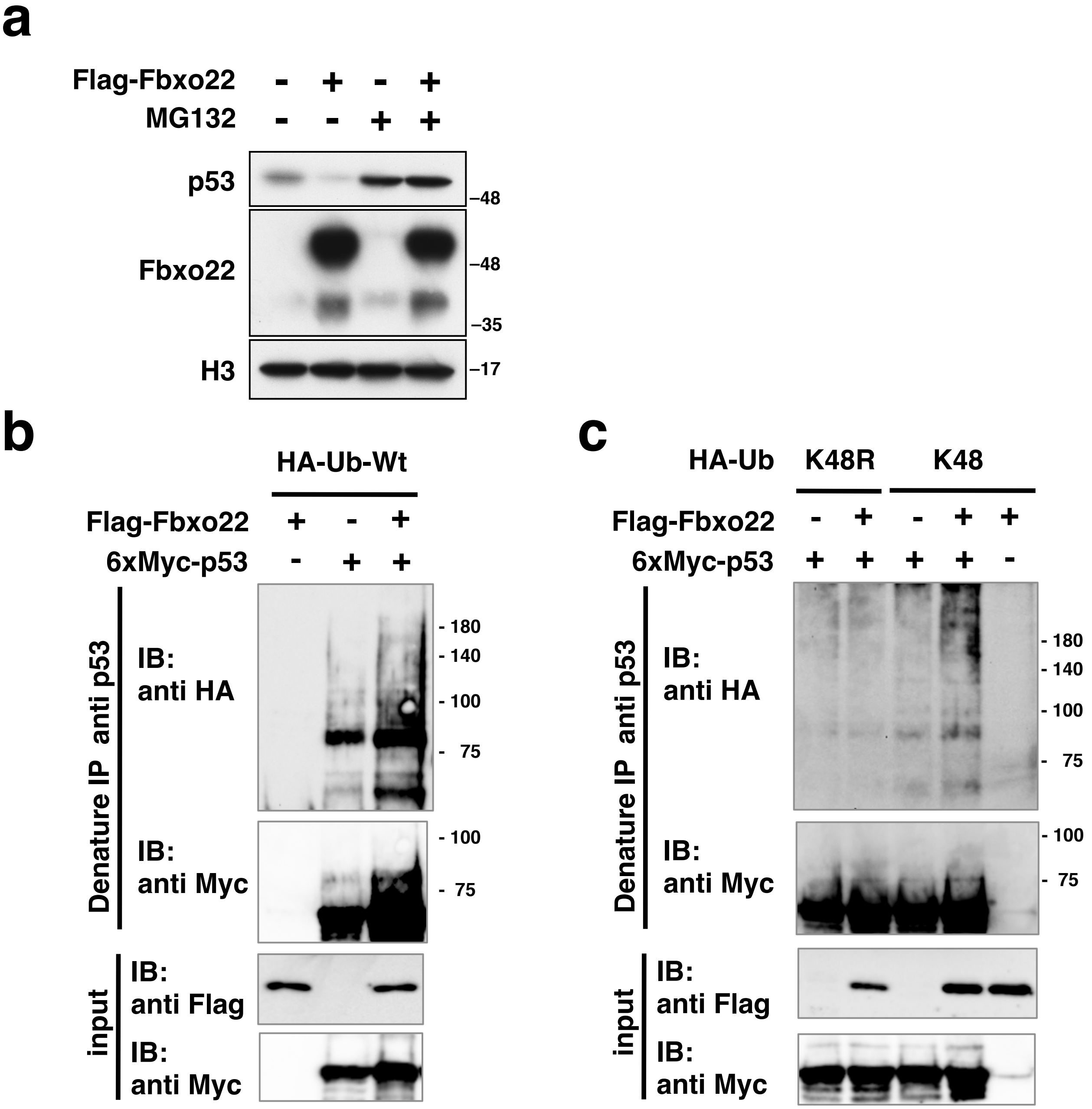


**Supplementary Figure 4 SCF$^{Fbxo22}$ promotes K48-linked ubiquitylation of p53**

**(a)** RPE cells expressing Dox-inducible Flag-Fbxo22 were treated with or without doxycyclin (1 μg/ml) for 48 hrs, and were then treated with or without MG132 (10 μg/ml) for 1 hr. The cell lysates were subjected to immunoblotting using the indicated antibodies. **(b)(c)** HEK293 cells were transfected with expression plasmids for 6xMyc-p53, Flag-Fbxo22, and HA-Ub-Wt **(b)**, HA-Ub-K48 or HA-Ub-K48R **(c)**, and were then incubated with MG132 (20 μg/ml) for 5 hrs. Total cell lysates were subjected to immunoprecipitation with anti-p53 antibody under denaturing conditions to prevent the detection of ubiquitylation of both E3 ligase itself and p53-associated proteins. The resultant precipitates and the lysates (Input) were subjected to immunoblotting using the indicated antibodies.

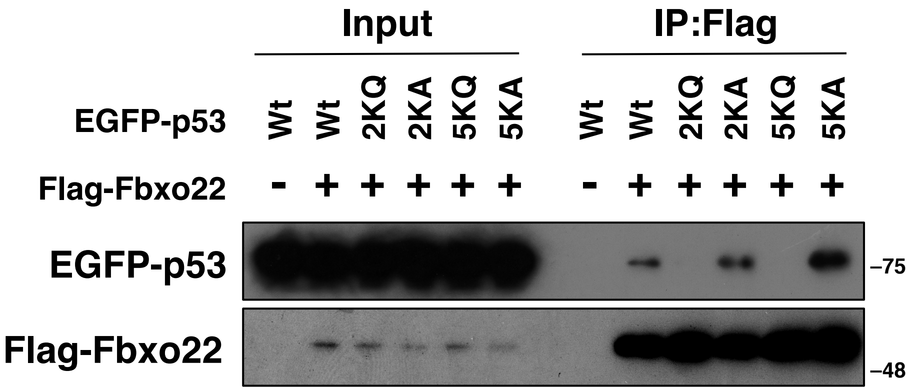


**Supplementary Figure 5 Fbxo22 binds to p53 mutants substituted lysine to alanine, but not those substituted lysine to glutamine, at two methylated or five acetylated sites**

HCT116 p53$^{-/-}$ cells expressing Dox-inducible Flag-Fbxo22 together with wild-type EGFP-p53 (Wt) or the indicated mutants of EGFP-p53 were incubated for 48 hrs in the presence or absence of doxycycline (1 μg/ml) and were then treated with MG132 (10 μg/ml) for 2 hrs. The lysates were immunoprecipitated using Flag M2 affinity gel. The resultant immunoprecipitates and lysates (Input) were subjected to immunoblotting using the indicated antibodies.

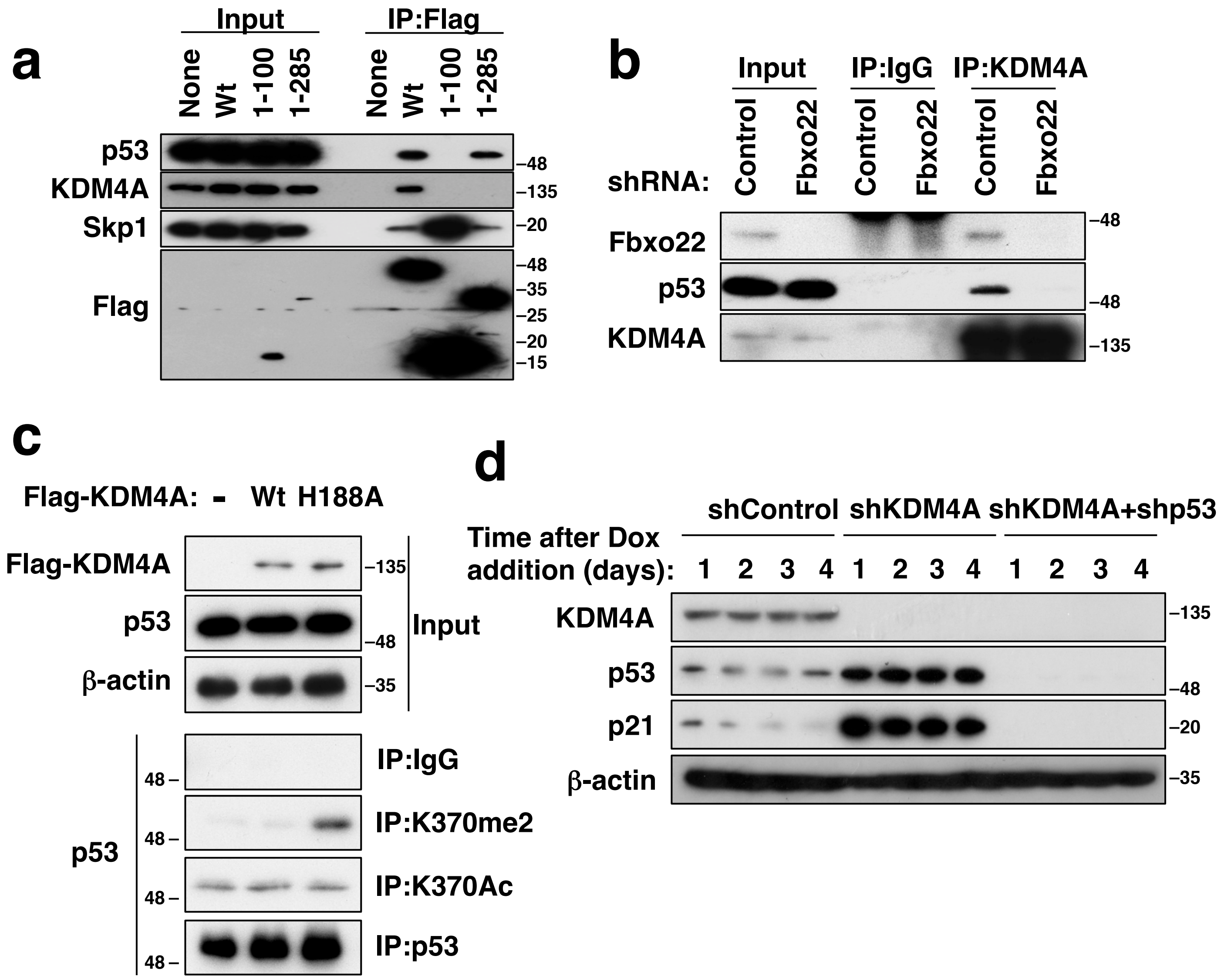


**Supplementary Figure 6 $SCF^{Fbxo22}$ forms a stable complex with KDM4A and p53**

**(a)** RPE cells expressing Dox-inducible full-length Flag-Fbxo22 (Wt), the indicated C-terminal truncated mutants were incubated for 48 hrs in the presence or absence of doxycycline (1 μg/ml) and were then treated with MG132 (10 μg/ml) for 2 hrs. Cells were then harvested and the lysates were subjected to immunoprecipitation using Flag M2 affinity gel. The resultant precipitates were subjected to immunoblotting using the indicated antibodies. **(b)** RPE cells expressing the Dox-inducible shControl or shFbxo22 were treated with doxycycline (1 μg/ml) for 48 hrs and were then treated with MG132 (10 μg/ml) for 2 hrs. Cells were then harvested and the lysates were immunoprecipiated with either a control IgG or anti-KDM4A antibodies. The resultant precipitates were subjected to immunoblotting using the indicated antibodies. **(c)** RPE cells expressing the Dox-inducible wild-type KDM4A (Wt) or its mutant (H188A) were treated with or without doxycycline (1 μg/ml) for 48 hrs and then treated with MG132 (10 μg/ml)for 2 hrs. The cell lysates were immunoprecipitated with a control IgG, anti-methylated p53 (K370me2), anti-acetylated p53 (K370Ac), or anti-p53 antibodies. The resultant immunoprecipitates and the lysates (Input) were subjected to immunoblotting using the indicated antibodies. **(d)** RPE cells as in Figure 5e were harvested at the indicated times and the lysates were subjected to immunoblotting using the indicated antibodies (see Figure 5e).

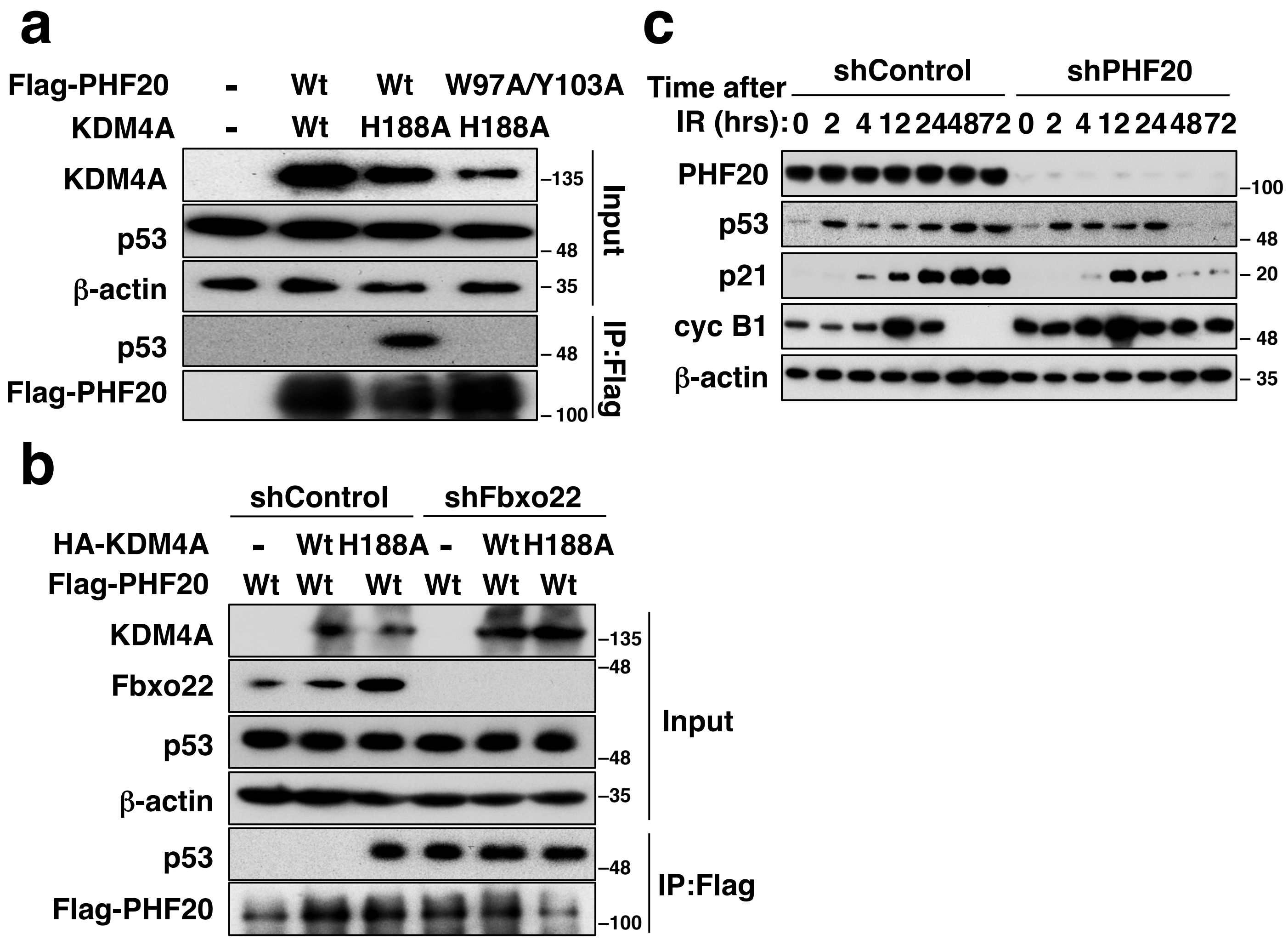


**Supplementary Figure 7 PHF20 protects methylated p53 from SCFFbxo22-targeted degradation**

**(a)** RPE cells expressing the Dox-inducible wild-type KDM4A (Wt) or its mutant (H188A), together with wild-type Flag-PHF20 (Wt) or its mutant (W97A/Y103A), were incubated for 48 hrs in the presence of doxycycline (1 μg/ml) and were then treated with MG132 (10 μg/ml) for 2 hrs. The lysates were immunoprecipitated using anti-Flag M2 affinity gel. The resultant immunoprecipitates and lysates (Input) were subjected to immunoblotting using the indicated antibodies. **(b)** RPE cells expressing the Dox-inducible shControl or shFbxo22, together with either wild-type HA-KDM4A (Wt) or its H188A mutant, and wild-type Flag-PHF20 were incubated for 48 hrs in the presence of doxycycline (1 μg/ml) and were then treated with MG132 (10 μg/ml) for 2 hrs. The lysates were immunoprecipitated using Flag M2 affinity gel and the resultant precipitates as well as lysates (Input) were subjected to immunoblotting using the indicated antibodies. **(c)** RPE cells expressing the Dox-inducible shControl or shPHF20 were treated with doxycycline (1 μg/ml) for 48 hrs and then with IR (6 Gy). Lysates of cells collected at the indicated times were subjected to immunoblotting using the indicated antibodies.

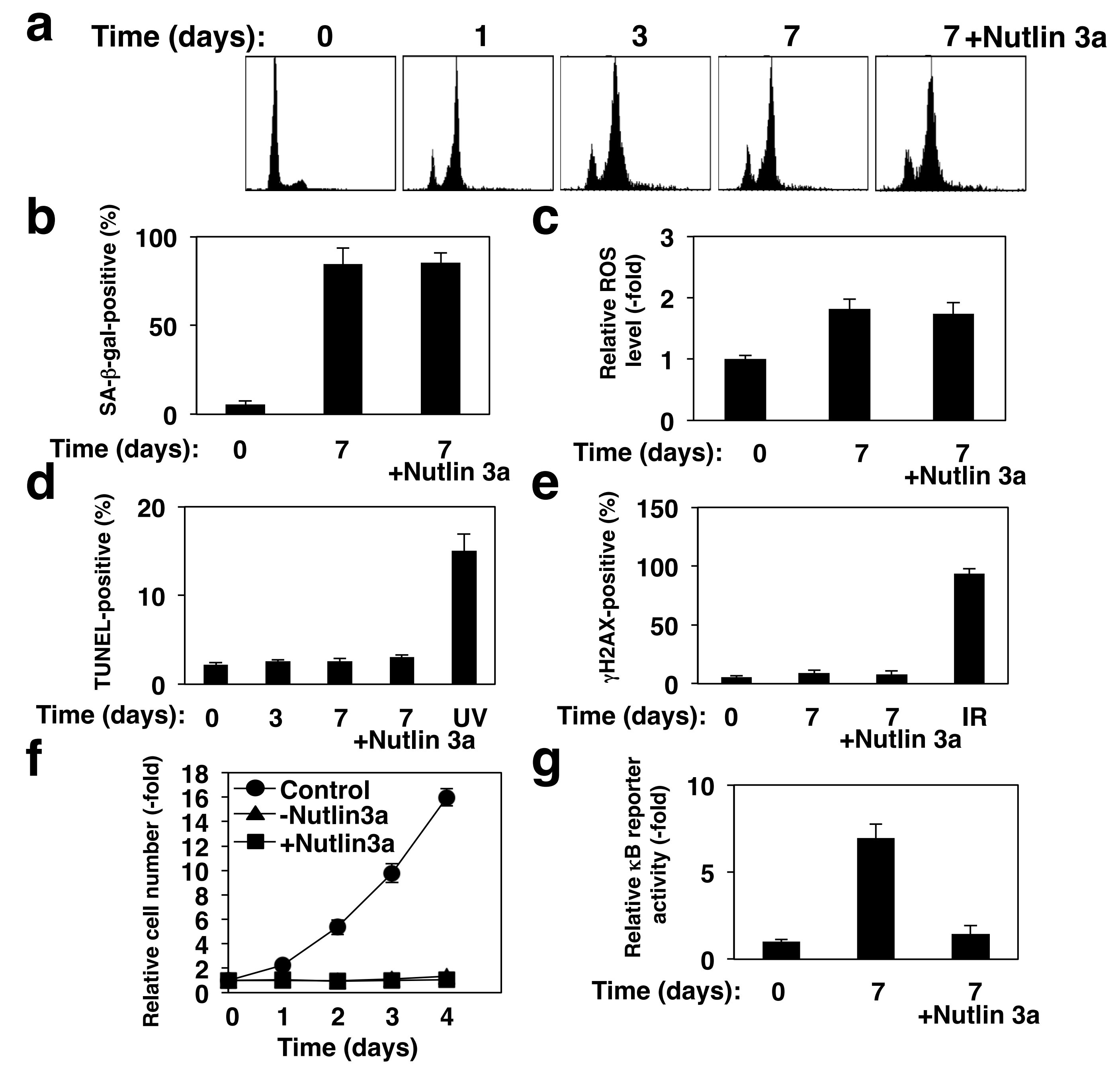


**Supplementary Figure 8 Nutlin 3a treatment according to the experimental outline as in Figure 6a effectively induces senescent phenotypes and prolonged treatment of Nutlin 3a does not affect most of the senescent phenotypes, but strongly suppresses κB reporter activity**

HCA2 cells were synchronized at G2 phase with RO3306 (9 μM)(Time:0), treated with 5 μM Nutlin-3a, and released into fresh medium in the presence or absence of doxycycline (1 μg/ml) at 3 days (see Figure 6a). Cells at the indicated times were subjected to FACS analysis **(a)**, an SA-β-gal assay **(b)**, and analysis of the cellular ROS level **(c)**. Cells treated as in **(a)** as well as those treated with UV-C (30 J/m2) or IR (10 Gy) were subjected to a TUNEL assay **(d)** or γH2AX staining **(e)** at the indicated times. Cell numbers of HCA2 cells treated as in **(a)** as well as asynchronous HCA2 cells as a control were determined **(f)**. **(g)** HCA2 cells expressing the NF-κB-luciferase reporter construct were treated as in **(a)**, and cell lysates at the indicated times were then analyzed for luciferase assay. Data presented as means ±s.d. of at least three independent experiments.

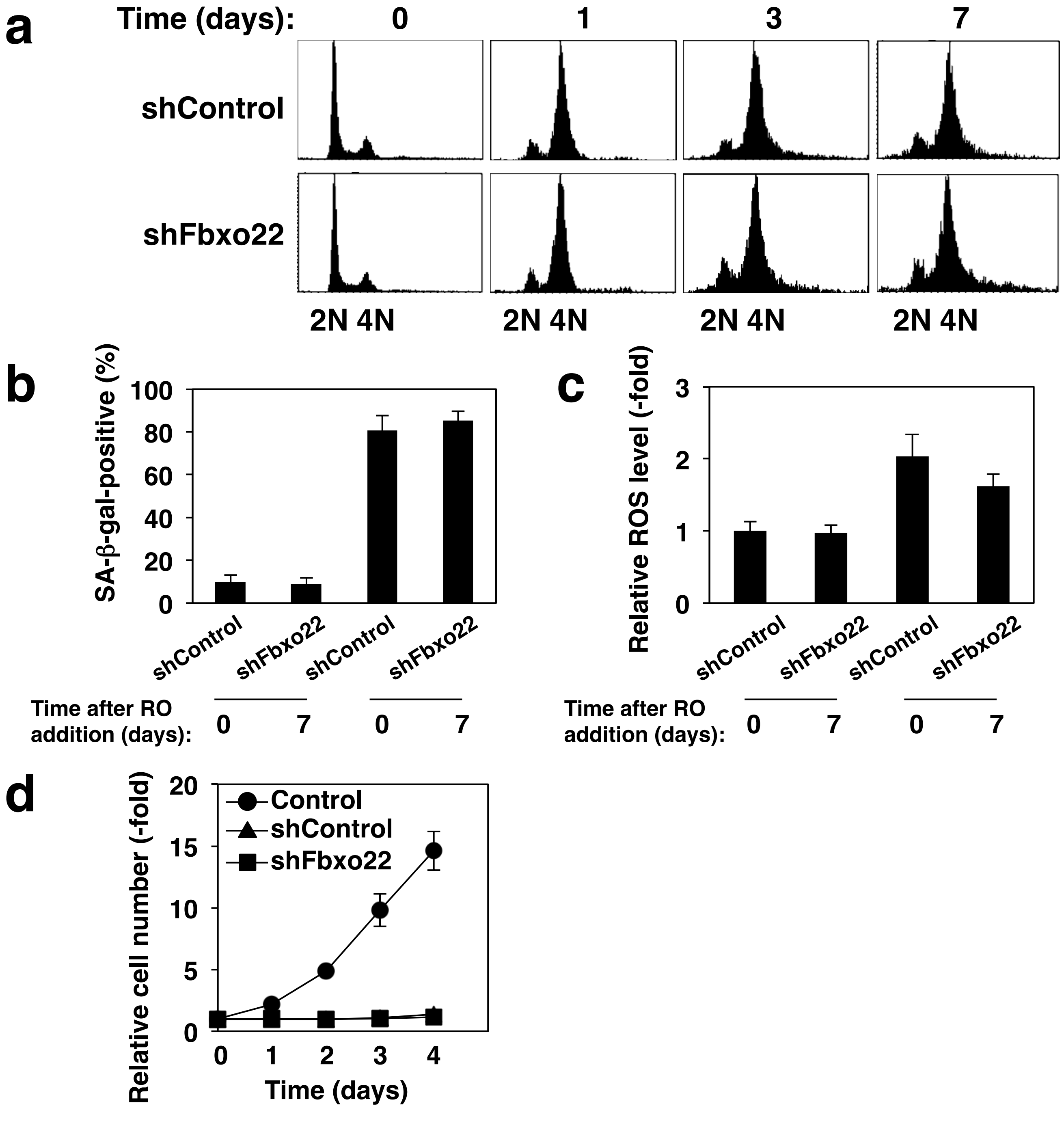


**Supplementary Figure 9 Fbxo22 depletion in Nutlin3a-induced senescent cells does not affect the cell cycle profile, SA-β-gal-positive cells, the ROS level, or cessation of cell proliferation**

HCA2 cells expressing the Dox-inducible shControl and shFbxo22 were synchronized at G2 phase with RO3306 (9 μM)(Time:0), treated with 5 μM Nutlin-3a, and released into fresh medium in the presence of doxycycline (1 μg/ml) at 3 days (see Figure 6a). Cells at the indicated times were subjected to FACS analysis **(a)**, an SA-β-gal assay **(b)**, and analysis of the cellular ROS level **(c)**. **(d)** Cell numbers of HCA2 cells treated as in **(a)** as well as asynchronous HCA2 cells expressing the Dox-inducible shControl in the presence of doxycycline (1 μg/ml) as a control were determined. Data presented as means ±s.d. of at least three independent experiments.

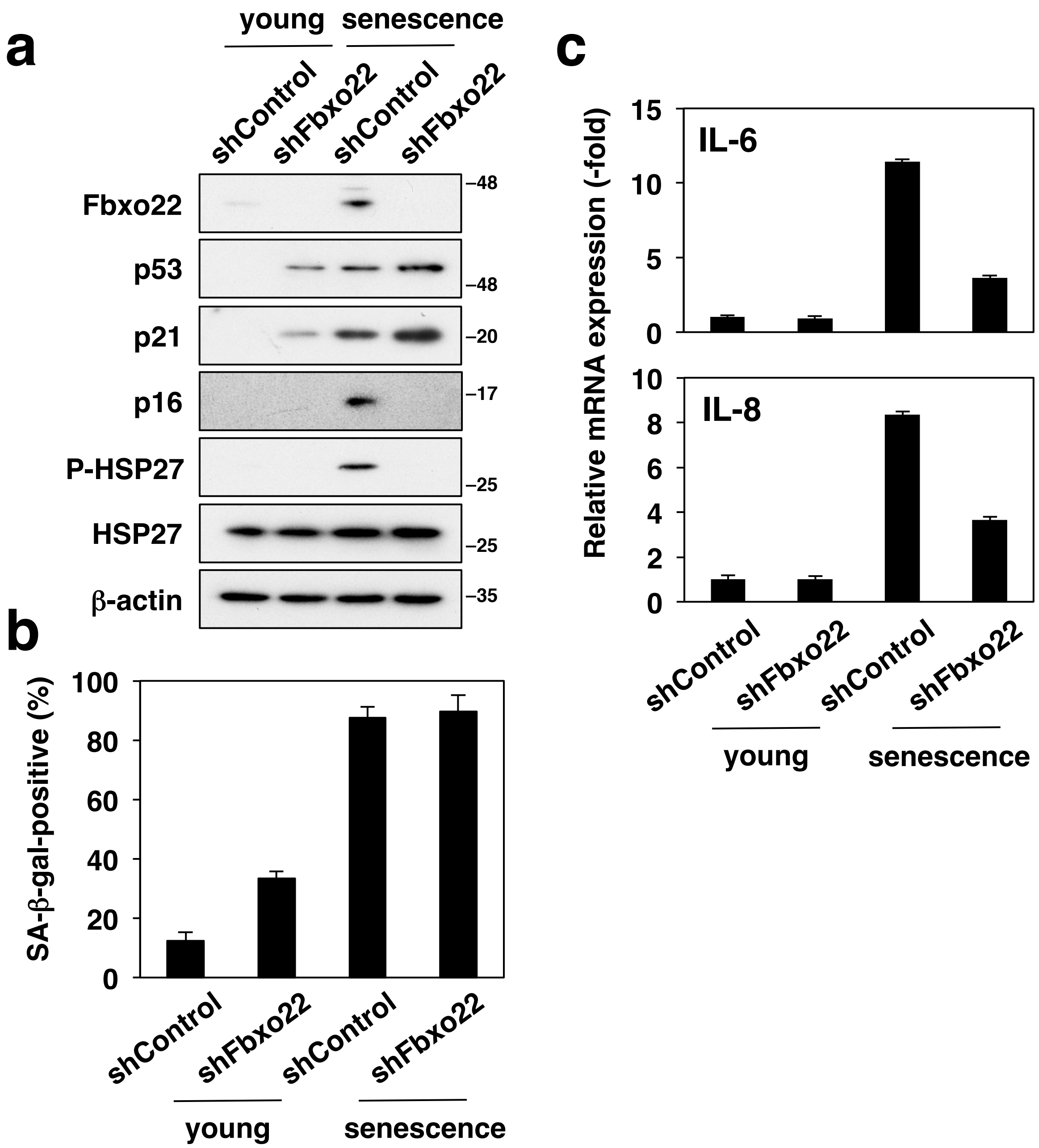


**Supplementary Figure 10 Fbxo22 depletion in replicative senescent cells suppresses induction of p16 and SASP, but does not affect SA-β-gal positivity**

Young (PD12) or senescent (PD>60) HCA2 cells expressing the Dox-inducible shControl and shFbxo22 were treated with doxycycline (1 μg/ml) at 3 days. Cells, cell lysates or total RNAs were then subjected to immunoblotting using the indicated antibodies **(a)**, an SA-β-gal assay **(b)**, and quantitative PCR analysis using IL-6 and IL-8 primers **(c)**. Data presented as means ±s.d. of at least three independent experiments.

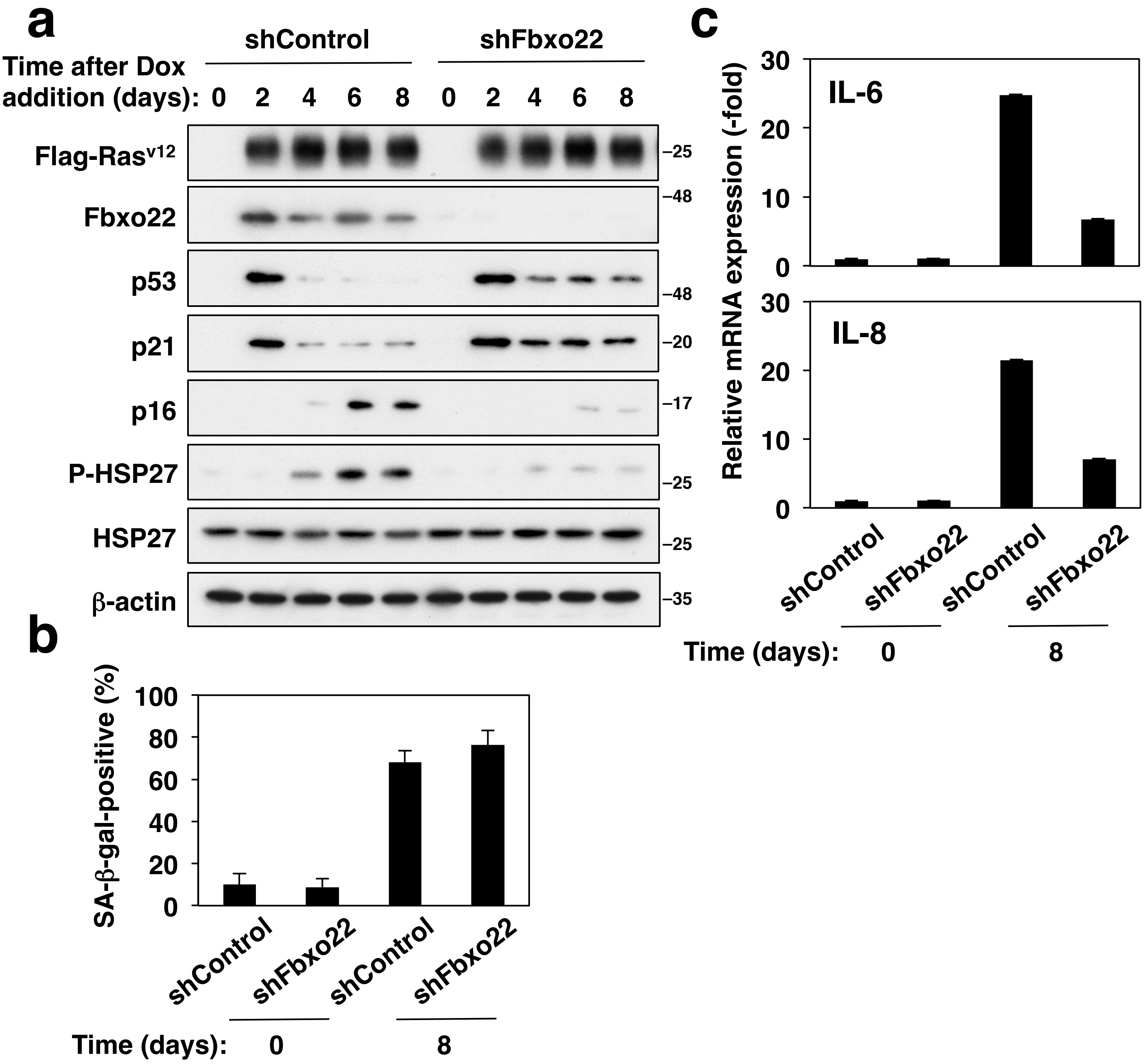


**Supplementary Figure 11 Fbxo22 depletion in Ras$^{V12}$-induced senescent cells suppresses induction of p16 and SASP, but does not affect SA-β-gal positivity**

HCA2 cells expressing the Dox-inducible Flag-H-RasV12 together with the shControl and shFbxo22 were treated with doxycycline (1 μg/ml). Cells, cell lysates or total RNAs at the indicated times (after addition of doxycycline) were subjected to immunoblotting using the indicated antibodies **(a)**, an SA-β-gal assay **(b)**, and quantitative PCR analysis using IL-6 and IL-8 primers **(c)**. Data presented as means ±s.d. of at least three independent experiments.

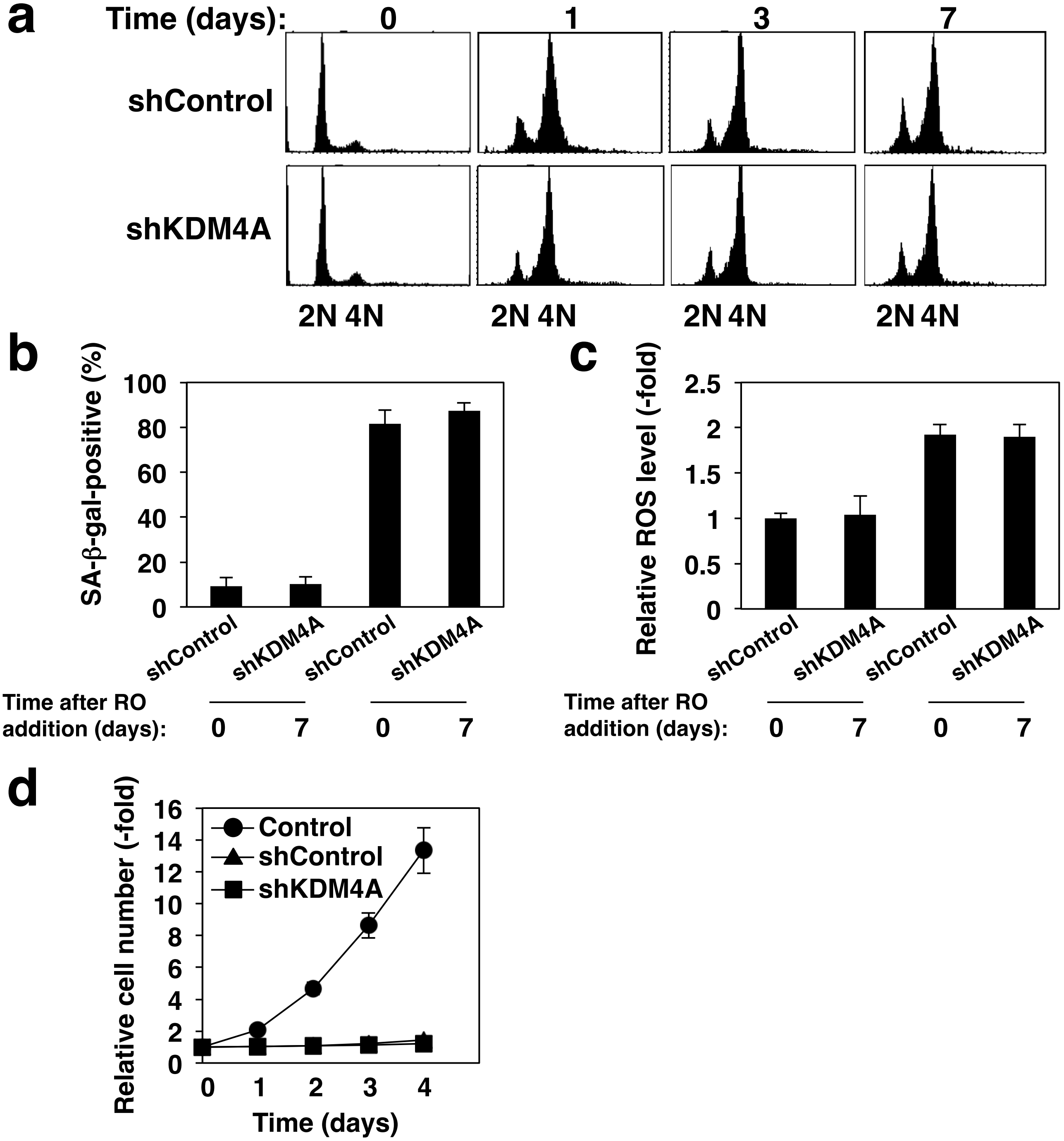


**Supplementary Figure 12 KDM4A depletion in Nutlin3a-induced senescent cells does not affect the cell cycle profile, SA-β-gal-positive cells, the ROS level, or cessation of cell proliferation**

HCA2 cells expressing the Dox-inducible shControl and shKDM4A were synchronized at G2 phase with RO3306 (9 μM)(Time:0), treated with 5 μM Nutlin-3a, and released into fresh medium in the presence of doxycycline (1 μg/ml) at 3 days (see Figure 6a). Cells at the indicated times were subjected to FACS analysis **(a)**, an SA-β-gal assay **(b)**, and analysis of the cellular ROS level **(c)**. **(d)** Cell numbers of HCA2 cells treated as in **(a)** as well as asynchronous HCA2 cells expressing the Dox-inducible shControl in the presence of doxycycline (1 μg/ml) as a control were determined. Data presented as means ±s.d. of at least three independent experiments.

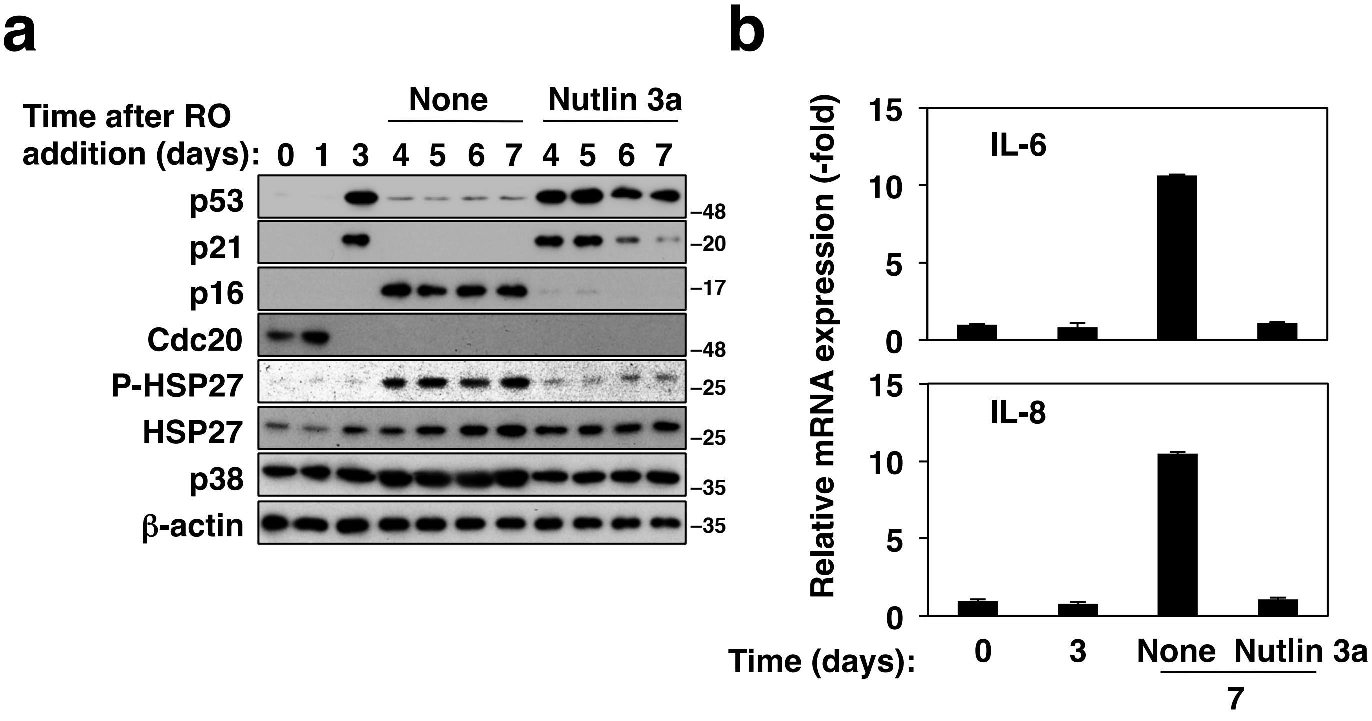


**Supplementary Figure 13 Prolonged activation of p53 inhibits induction of p16 and SASP in senescent cells**

HCA2 cells were synchronized at G2 phase with RO3306 (9 μM)(Time:0), treated with 5 μM Nutlin-3a, and released into fresh medium at 3 days in the presence or absence (None) of 5 μM Nutlin-3a (see Figure 6a). Cell lysates or total RNAs from cells collected at the indicated times were subjected to immunoblotting using the indicated antibodies **(a)** or qPCR analysis using IL-6 and IL-8 primers **(b)**, respectively. Data presented as means ±s.d. of at least three independent experiments.

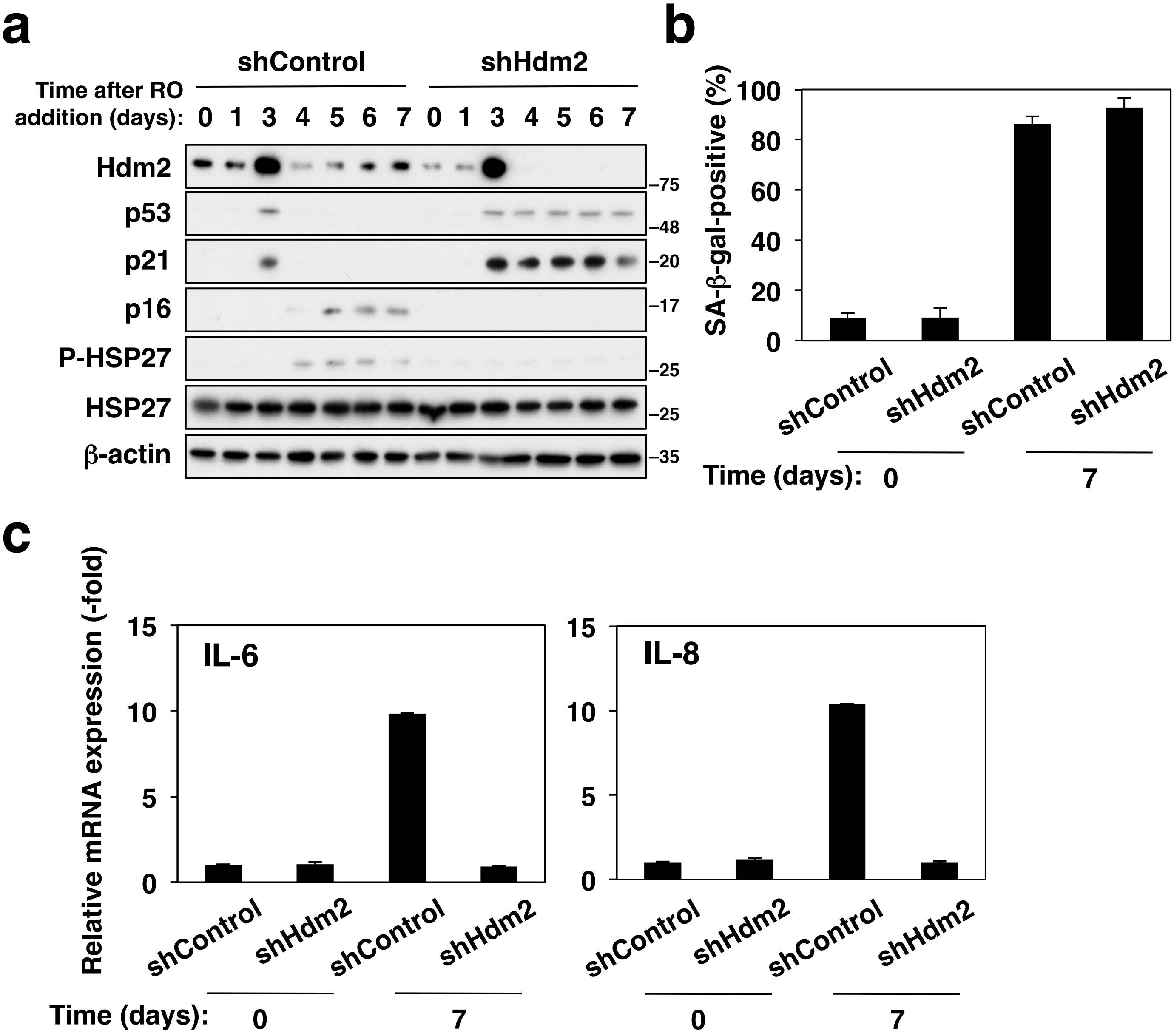


**Supplementary Figure 14 Hdm2 depletion in senescent cells suppresses induction of p16 and SASP**

HCA2 cells expressing the Dox-inducible shControl and shHdm2 were synchronized at G2 phase with RO3306 (9 μM)(Time:0), treated with 5 μM Nutlin 3a, and released into fresh medium with doxycycline (1 μg/ml) at 3 days (see Figure 6a). Cells, cell lysates or total RNAs from cells collected at the indicated times were subjected to immunoblotting using the indicated antibodies **(a)**, an SA-β-gal assay **(b)** or qPCR analysis using IL-6 and IL-8 primers **(c)**, respectively. Data presented as means ±s.d. of at least three independent experiments.

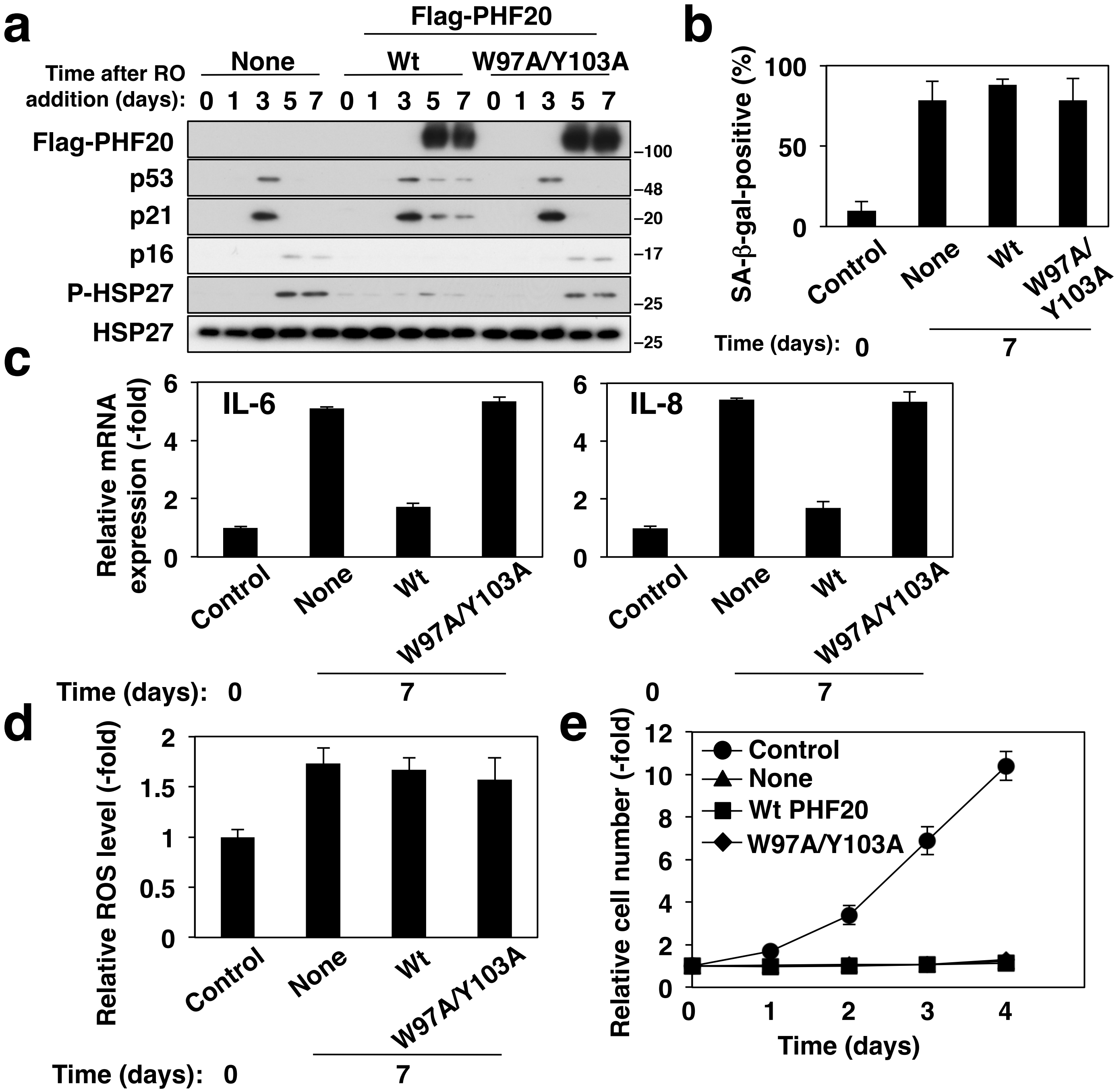


**Supplementary Figure 15 Expression of wild-type PHF20 in Nutlin 3a-induced senescent cells suppresses induction of p16 and SASP, but does not affect SA-β-gal positivity, the cellular ROS level, or cessation of cell proliferation**

HCA2 cells (None) or cells expressing the Dox-inducible wild-type (Wt) or mutant (W97A/Y103A) Flag-PHF20 were synchronized at G2 phase with RO3306 (9 μM)(Time: 0), treated with 5 μM Nutlin 3a, and released into fresh medium with doxycycline (1 μg/ml) at 3 days (see Figure 6a). Cells, cell lysates or total RNAs at the indicated times (after addition of doxycycline) were subjected to immunoblotting using the indicated antibodies **(a)**, an SA-β-gal assay **(b)**, quantitative PCR analysis using IL-6 and IL-8 primers **(c)**, and analysis of the cellular ROS level **(d)**. **(e)** The relative number of HCA cells (None) or cells expressing as in **(a)** were detemined at the indicated times. Cell numbers of asynchronous HCA2 cells were used for comparison. Data presented as means ±s.d. of at least three independent experiments.

**a**

```
                     171                          Exon 1                                219
                      ↓                                                                  ↓
Wild-type        Wt        CACCTACGTGCTGAGTAACCTTGCGGAGGTGGTGGAGCGTGTGTTTACC
                 Wt        CACCTACGTGCTGAGTAACCTTGCGGAGGTGGTGGAGCGTGTGTTTACC

Heterozygous     Wt        CACCTACGTGCTGAGTAACCTTGCGGAGGTGGTGGAGCGTGTGTTTACC
                 Δ181-194  CACCTACGTG              GATGGTGGTGGAGCGTGTGTTTACC

*
Nullizygous 1    Δ190-195  CACCTACGTGCTGAGTAAC      GAGGTGGTGGAGCGTGTGTTTACC
                 Δ181-211  CACCTACGTG                                TGTTTACC

*
Nullizygous 2    Δ194-195  CACCTACGTGCTGAGTAACCTTG  GAGGTGGTGGAGCGTGTGTTTACC
                 Δ194-195  CACCTACGTGCTGAGTAACCTTG  GAGGTGGTGGAGCGTGTGTTTACC

**
Nullizygous 3    Δ194-197  CACCTACGTGCTGAGTAACCTTG    GGTGGTGGAGCGTGTGTTTACC
                 Δ194-197  CACCTACGTGCTGAGTAACCTTG    TGTGGTGGAGCGTGTGTTTACC
```

**b**

| Genotype | Body weight (g) | Brain | | Heart | | Lung | | Liver | | Kidney | | Spleen | | Thymus | |
|---|---|---|---|---|---|---|---|---|---|---|---|---|---|---|---|
| | | (g) | Relative (/ 100g B.W.) | (g) | Relative (/ 100g B.W.) | (g) | Relative (/ 100g B.W.) | (g) | Relative (/ 100g B.W.) | (g) | Relative (/ 100g B.W.) | (g) | Relative (/ 100g B.W.) | (g) | Relative (/ 100g B.W.) |
| wild | 27.8 | 0.48 | 1.73 | 0.15 | 0.54 | 0.16 | 0.58 | 1.33 | 4.78 | 0.39 | 1.40 | 0.13 | 0.47 | 0.05 | 0.18 |
| wild | 27.41 | 0.48 | 1.75 | 0.15 | 0.55 | 0.16 | 0.58 | 1.43 | 5.22 | 0.4 | 1.46 | 0.13 | 0.47 | 0.04 | 0.15 |
| hetero | 29.4 | 0.47 | 1.60 | 0.14 | 0.48 | 0.15 | 0.51 | 1.34 | 4.56 | 0.36 | 1.22 | 0.1 | 0.34 | 0.05 | 0.17 |
| homo | 16.4 | 0.39 | 2.38 | 0.1 | 0.61 | 0.11 | 0.67 | 0.8 | 4.88 | 0.23 | 1.40 | 0.06 | 0.37 | 0.06 | 0.37 |
| homo | 18.47 | 0.39 | 2.11 | 0.09 | 0.49 | 0.1 | 0.54 | 0.83 | 4.49 | 0.23 | 1.25 | 0.05 | 0.27 | 0.03 | 0.16 |

**Supplementary Figure 16 Sequence alignment of mutant alleles with Fbxo22 exon 1 generated by CRISPR/CAS9**

**(a)** Mutant alleles of one *Fbxo22*$^{+/-}$ and three *Fbxo22*$^{-/-}$ mice. Mutated bases are indicated in red. * used for histo-pathological analyses, ** died within two days. **(b)** Body and organ weights of mice with the indicated genotypes.

Exon6-239 ↓ Intron7

**Tcaf2** (NM_146174) -CTCAGCCGGTGGGTGCTTCAGGG

Exon1-177 ↓ Exon1-199 ↓

**DBF4** (NM_001190717) -CCCAGCATGTAGGTGCCTGGTGG

Exon1-137 ↓ (anti-sense) Exon1-115 ↓

**Caf1, subunit B (p60)** (NM_028083) -CTCTGCACGCAGGTGCATAGCAG

Exon1-328 ↓ (anti-sense) Exon1-306 ↓

**Mtus2** (NM_029920) –CTCCGCACGCAGCTCCTTCGCAG

Exon8-44 ↓ (anti-sense) Exon8-22 ↓

**Cyp4a10** (NM_010011) –CTCAGCACGTAGGTCCTTGTCAG

Exon8-44 ↓ (anti-sense) Exon8-22 ↓

**Cyp4a32** (NM_001100181) -CTCAGCACGTAGGTCCTTGTCAG

Exon11-437 ↓ (anti-sense) Exon11-415 ↓

**Pafah2** (NM_133880) -CTCAGCACATAAGTACTTCATAG

Exon7-44 ↓ (anti-sense) Exon7-22 ↓

**Cyp4a31** (NM_001252539) -CTCAGCACGAAGGTCCTTGTCAG

Exon11-101 ↓ (anti-sense) Exon11-79 ↓

**Cdc123** (NM_133837) -CTCACCACGTAGGTTGTTCTCAG

**Supplementary Figure 17 Off target sequences predicted by the recognition rule**

All nine potential off-target loci predicted by the recognition rule described previously[30] were amplified by PCR. We confirmed that there were no deletions, insertions or mutations within these loci. The intron sequence is indicated in red.

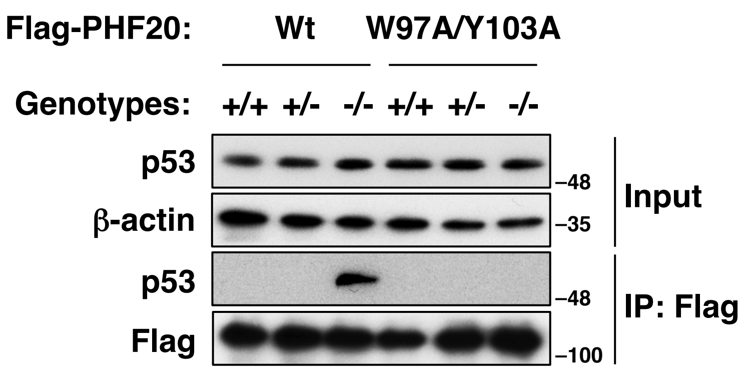


**Supplementary Figure 18 Wild-type PHF20, but not its W97A/Y103A mutant, formed a complex with p53 in MEFs from Fbxo22$^{-/-}$ mice.**

Primary MEFs with the indicated genotypes expressing Dox-inducible wild-type Flag-PHF20 (Wt) or its mutant (W97A/Y103A) were incubated for 48 hrs in the presence of doxycycline (1 μg/ml) and were then treated with MG132 (10 μg/ml) for 2 hrs. The cell lysates were immunoprecipitated using anti-Flag M2 affinity gel. The resultant immunoprecipitates and lysates (Input) were subjected to immunoblotting using the indicated antibodies.

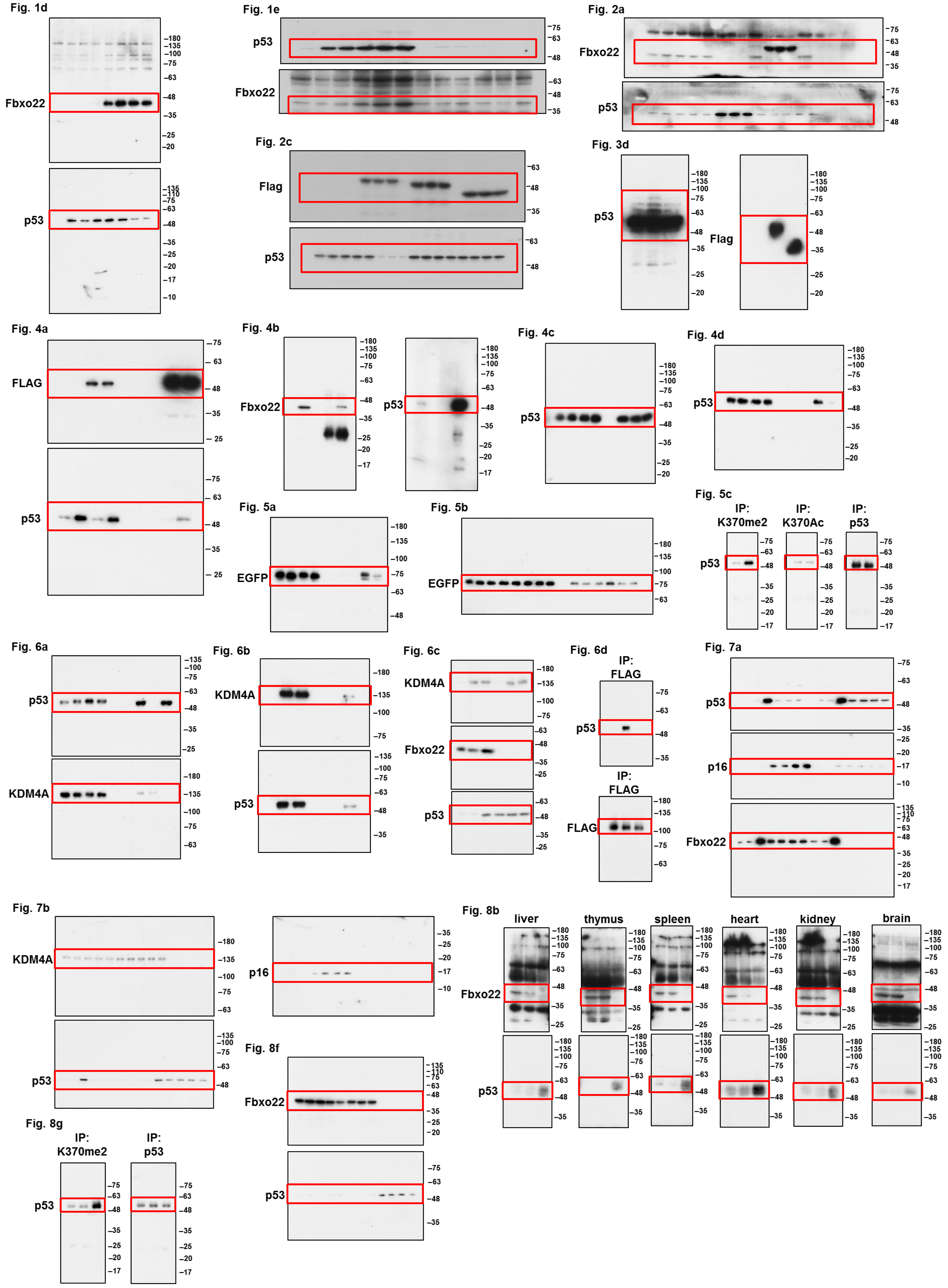


**Supplementary Figure 19 Full scans of the most important blots**

## Supplementary Table 1. shRNA sequences used in this study

| Target gene | Sequence | Reference | Type |
|---|---|---|---|
| Fbxo22 | GGAATTGTAGTGACTCCAATG | this study | lentivirus |
| KDM4A | GGACTTAGCTTCATAACT | (Pedersen et al., 2014) | lentivirus |
| Luciferase | CGTACGCGGAATACTTCG | (Elbashir et al., 2001) | lentivirus |
| PHF20 | AAGAGGATGGATCTTCTGAAT | (Cui et al., 2012) | lentivirus |
| p21 | GGTGACTTCGCCTGGGAGCGT | (Zhang et al., 2005) | lentivirus |
| p53 | ACTCCAGTGGTAATCTACT | (Hong et al., 2009) | lentivirus |

## Supplementary Table 2. Antibodies used in this study

| Antibodies | Species | dilution | Source |
|---|---|---|---|
| Anti-beta-Actin (6276) | Mouse | 1/10000 | Abcam, Cambridge, United Kingdom |
| Anti-DARC (ab40821) | Goat | 1/1000 | Abcam, Cambridge, United Kingdom |
| Anti-EGFP (D153-3) | Rat | 1/5000 | MBL, Nagoya, Japan |
| Anti-Fbxo22 (FF-7) | Mouse | 1/200 | Santa Cruz Biotechnologies, Santa Cruz, CA |
| Anti-Fbxo22 (N3C3) | Rabbit | 1/2000 | GeneTex, Irvine, CA |
| Anti-FLAG (M2) | Mouse | 1/2000 | Sigma, St. Louis, MO |
| Anti-HA (3F10) | Rat | 1/2000 | Roche, Basel, Switzerland |
| Anti-Histone H3 triMe K4 (07-473) | Rabbit | N/A | MIlipore, Billerica, MA |
| Anti-Histone H3 (ab1791) | Rabbit | 1/10000 | Abcam, Cambridge, United Kingdom |
| Anti-Histone H2AX P-S139 (GTX11174) | Rabbit | 1/2000 | GeneTex, Irvine, CA |
| Anti-HSP27-P-S82(D1H2) | Rabbit | 1/2000 | Cell Signaling Technology, Boston, MA |
| Anti-HSP27 (G31) | Mouse | 1/2000 | Cell Signaling Technology, Boston, MA |
| Anti-KDM4A (A300-861A) | Rabbit | 1/1000 | Bethyl Laboratories, Montgomery, TX |
| Anti-KDM4A (N154/32) | Mouse | 1/200 | UC Davis/NIH NeuroMab Facility, Davis, CA |
| Anti-Myc (9E10) | Rabbit | 1/500 | Santa Cruz Biotechnologies, Santa Cruz, CA |
| Anti-PHF20 (D96F6) | Rabbit | 1/2000 | Cell Signaling Technology, Boston, MA |
| Anti-PPP2R5C (H-40) | Rabbit | 1/1000 | Santa Cruz Biotechnologies, Santa Cruz, CA |
| Anti-p16 (JC8) | Mouse | 1/500 | Santa Cruz Biotechnologies, Santa Cruz, CA |
| Anti-p16 (M-156) | Mouse | 1/200 | Santa Cruz Biotechnologies, Santa Cruz, CA |
| Anti-p21 (F-5) | Mouse | 1/200 | Santa Cruz Biotechnologies, Santa Cruz, CA |

## Supplementary Table 2. Antibodies used in this study (Continued)

| Antibodies | Species | dilution | Source |
|---|---|---|---|
| Anti-p53 (NCL-p53-505) | Mouse | 1/1000 | Leica Biosystems, Nussloch, Germany |
| Anti-p53 (DO-1) | Mouse | 1/500 | Santa Cruz Biotechnologies, Santa Cruz, CA |
| Anti-p53 Ac-K370 (Ab183544)) | Rabbit | 1/200 | Abcam, Cambridge, United Kingdom |
| Anti-p53 P-S15 (9284) | Rabbit | 1/1000 | Cell Signaling Technology, Boston, MA |
| Anti-p53 diMe-K370 (ATB-H0007) | Rabbit | 1/500 | Ameritech Biomedicines, Houston, TX |
| Anti-WIPI (F-6) | Mouse | 1/100 | Santa Cruz Biotechnologies, Santa Cruz, CA |
| Anti-Ubiquitin (FK2) | Mouse | 1/1000 | Enzo Life Science, New York, NY |
| Anti-Histone H3 monoMe K4 | Mouse | N/A | Provided by Dr. Hiroshi Kimura |
| Anti-Histone H3 Ac-K27 | Mouse | N/A | Provided by Dr. Hiroshi Kimura |
| Anti-P-RNA pol II | Mouse | N/A | Provided by Dr. Hiroshi Kimura |
